# Effective recognition of double-stranded RNA does not require activation of cellular inflammation

**DOI:** 10.1126/sciadv.ads6498

**Published:** 2025-04-09

**Authors:** Karolina Drazkowska, Julia Cieslicka, Michal Kitowicz, Anna Pastucha, Lukasz Markiewicz, Wiktoria Szymanek, Krzysztof Goryca, Tomasz Kowalczyk, Dominik Cysewski, Andreas R. Bausch, Pawel J. Sikorski

**Affiliations:** ^1^ Laboratory of Epitranscriptomics, Faculty of Biology, Biological and Chemical Research Centre, University of Warsaw, Warsaw, Poland.; ^2^ Center for Functional Protein Assemblies, Technical University of Munich, Munich, Germany.; ^3^ Centre of New Technologies, University of Warsaw, Warsaw, Poland.; ^4^ Genomics Core Facility, Centre of New Technologies, University of Warsaw, Warsaw, Poland.; ^5^ Clinical Research Centre, Medical University of Bialystok, Bialystok, Poland.

## Abstract

Excess double-stranded RNA (dsRNA) is present in the cytoplasm of human cells, usually following viral infections. Recognition of dsRNAs activates innate immune pathways, leading to cellular inflammation and inhibition of cell growth. Here, we show that an effective dsRNA response may occur without the onset of inflammation. Pro-inflammatory [RLR (retinoic acid–inducible gene I–like receptor)–dependent pathway] and cell growth inhibitory mechanisms [oligoadenylate synthetase (OAS)/ribonuclease L (RNase L)– and dsRNA-activated protein kinase (PKR)–dependent pathways] can act independently. We found that the 5′ ends of dsRNA direct the onset of cellular inflammation, whereas the RNA duplex activates the OAS/RNase L and PKR pathways. Unexpectedly, three of the most common human RNA epitranscriptomic marks—i.e., *N*6-methyladenosine, 5-methylcytosine, and pseudouridine—had almost no influence on the immunogenicity of dsRNA; however, the presence of *N*6-methyladenosine inhibited the OAS/RNase L pathway. Our observations demonstrate how precisely innate immunity is fine tuned in cells to take appropriate countermeasures when a specific threat arises.

## Teaser

Effective recognition of double-stranded RNA in human cells does not require activation of the antiviral interferon pathway.

## INTRODUCTION

Double-stranded RNA (dsRNA) in human cells originates from endogenous (self-derived) and exogenous sources (*1*, *2*). Healthy human cells growing under optimal conditions have low levels of dsRNAs derived from, for example, the RNA interference pathway or those that are related to the presence of long noncoding RNA and genomic transposable elements. However, under some pathological conditions, dysregulation of the RNA machinery leads to the accumulation of excessive amounts of dsRNA (*1*, *3*–*8*). Viral infection is the primary source of dsRNA in human cells. In such situations, the dsRNA present in the host cell cytoplasm may be either an intermediate product of viral replication or the viral genome itself; some viral genomes consist of dsRNA (*2*, *9*). Therefore, human cells have evolved intricate mechanisms to recognize and respond to the presence of dsRNA as a potential threat of viral infection. This recognition is critical for triggering the human innate immune response, which is the first line of defense against viral infections.

Upon recognition of dsRNA, the host cell initiates an antiviral response aimed at limiting viral replication as much as possible and alerting neighboring uninfected cells of viral threats. In the cytoplasm of the host cell, dsRNAs are recognized by retinoic acid–inducible gene I (RIG-I)–like receptors (RLRs), which activate inflammatory signaling pathways through the mitochondrial antiviral signaling (MAVS) adaptor protein, leading to the production of interferons and cytokines and the activation of interferon-stimulated genes (ISGs) (*10*–*13*). In addition, host cells attempt to limit viral replication by slowing down their own metabolism and sacrificing their well-being. Two main mechanisms inhibit host cell growth upon viral infection: dsRNA-activated protein kinase (PKR) and oligoadenylate synthetase (OAS)/ribonuclease L (RNase L)–dependent pathways. Activation of the host PKR leads to a global shutdown of protein biosynthesis, resulting in reduced viral and host protein production (*14*–*16*). In contrast, viral and host single-stranded transcripts are degraded upon activation of the OAS/RNase L pathway (*17*–*19*). Host sensors differ in their mechanism of dsRNA recognition, recognizing specific dsRNA features or simply detecting the presence of a duplex of ribonucleic acids. RIG-I recognizes dsRNA on the basis of the dsRNA 5′ end structure and has the highest affinity for triphosphorylated dsRNA (ppp-dsRNA) (*11*, *13*, *20*–*22*). It was thought that the presence of the cap structure was sufficient to substantially decrease the affinity of RIG-I for dsRNA, but dsRNA with cap0 had similar affinity for dsRNA as the triphosphorylated counterpart, and only 2′-*O*-methylation of the cap structure abolishes the RIG-I binding to dsRNA (*20*). Another member of the RLR family, melanoma differentiation–associated protein 5 (MDA5), recognizes long duplex structures within transcripts rather than 5′ end modifications present on dsRNAs (*23*, *24*). Other reports showed that 5′ end modifications of dsRNA may play a role in its recognition by MDA5 (*12*, *25*). However, the exact mechanism by which MDA5 senses dsRNA is not yet fully understood. In the case of OAS1/2/3 and PKR, the presence of the RNA duplex seems to be the only requirement to classify the transcripts as a potential threat to the host cell (*15*, *26*, *27*).

Viruses, in the course of evolution, have acquired mechanisms that allow them to shield their nucleic acids from recognition by host sensors (*2*, *9*). To avoid recognition by receptors that sense the 5′ end of dsRNA, viral RNA (vRNA) can be capped like endogenous human transcripts (*28*). The cap structure not only prevents RIG-I from binding to the vRNA but also allows viruses to benefit from cap-dependent translation. In addition, vRNAs could undergo posttranscriptional modifications, similar to those observed in host transcripts, to mimic endogenous RNA molecules as closely as possible and further evade immune recognition (*29*). Among the modifications that have been identified in vRNA are those that are most abundant in the human transcriptome, namely *N*6-methyladenosine (m^6^A), pseudouridine (Ψ), and 5-methylcytosine (m^5^C).

Although viral strategies to evade immune recognition have been known for many years and the innate immune pathways leading to the activation of antiviral responses and inhibition of host cell growth have been thoroughly characterized, we still do not know how each feature of dsRNA affects host cell defense mechanisms. Does the mere presence of the RNA duplex, regardless of how it is modified, activate all the innate immune pathways of the host cell, or is each specific feature of the dsRNA responsible for activating a particular defense mechanism? Is it possible that, under certain conditions, these pathways are so independent of each other that dsRNA can activate only some of them? Does dsRNA recognition always lead to the activation of all possible defense mechanisms? Studies on the response of human cells to viral infection have always led to the conclusion that the presence of vRNA activates all possible defense mechanisms to fight the infection (*2*, *9*). A similar answer is provided by studies using a synthetic polyinosinic-polycytidylic acid compound, better known as poly(I:C), which is widely used to mimic viral infection (*27*, *30*–*35*). More detailed information about the function of individual host sensors comes from in vitro studies; however, on the basis of these results, it is difficult to understand how the innate defense system as a whole functions and how it is regulated.

Herein, we used an in vitro–prepared dsRNA carrying various modifications both at the 5′ end and in its body, or combinations thereof, to truly characterize host cell-dsRNA interplay. The use of properly prepared and modified dsRNAs allowed us to understand how this class of transcripts activated the innate immune pathway. We revealed that the activation of the immune pathway leading to the inhibition of cell growth can be achieved without the onset of cell inflammation. We showed that modifications at the 5′ end of dsRNA only affect the MAVS-dependent antiviral and inflammatory signaling pathway. Furthermore, the activation of this pathway is predominantly mediated by RIG-I, at least in the human cell lines tested. In contrast, the activation of the OAS/RNase L and PKR pathways, leading to cell growth inhibition, is exclusively dependent on the detection of ribonucleic acid duplexes, and their activation does not lead to interferon production. In addition, we outlined the extent to which epitranscriptomic marks affect innate immune pathways and found that m^6^A modification in the body of transcripts can protect dsRNAs from recognition by OAS proteins, whereas none of the tested modifications affected dsRNA recognition by PKR. Notably, OAS proteins, unlike PKR, appear to be more sensitive to RNA duplex relaxation, as the presence of m^6^A, but not Ψ or m^5^C, reduces the melting temperature of dsRNA.

## RESULTS

### The cap structure drives transcript immunogenicity

**Fig. 1.**
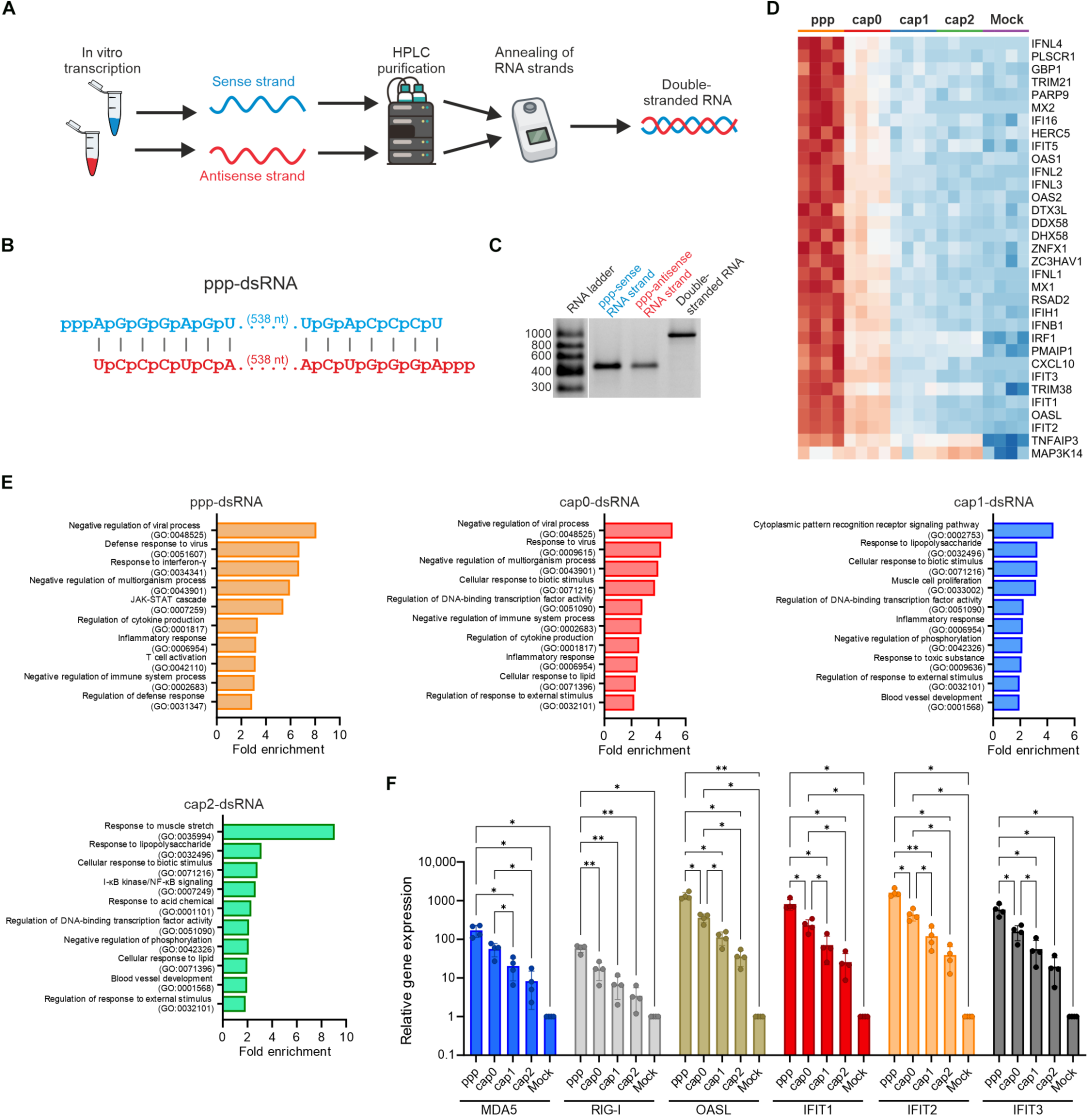
2′-*O*-Methylation of the cap structure reduces dsRNA immunogenicity. (**A**) Schematic of dsRNA generation: Complementary RNA strands are transcribed with T7 RNA polymerase, purified by HPLC. In the case of capped transcript generation, the RNA is enzymatically treated to remove uncapped RNA (this step is not shown in the scheme). Last, a pair of complementary strands is heated and slowly cooled down to force duplex formation, i.e., dsRNA. (**B**) Representation of in vitro–transcribed dsRNA with a triphosphate group at the 5′ end. Other 5′ end modifications are shown in fig. S1A. (**C**) Agarose gel analysis of sense and antisense strands (triphosphorylated 5′ ends) and annealed dsRNA. Additional analyses of modified dsRNAs are shown in fig. S1B. (**D**) Heatmap of differentially expressed genes (GO:0051607, “Defense response to virus”) in A549 cells transfected with 5′-modified dsRNAs (ppp-, cap0-, cap1-, and cap2-dsRNA). The full heatmap is shown in fig. S3. (**E**) Top 10 overrepresented GO terms for transfected A549 cells. (**F**) RT-qPCR validation of RNA-seq data: relative gene expression (means ± SD, *n* = 4). Statistical significance: *P < 0.05 and **P < 0.01 (one-way ANOVA with Tukey’s multiple comparisons test). Only statistically significant differences are shown.

The most widely used synthetic molecule to mimic viral infection and trigger the innate immune system is the poly(I:C) molecule (*27*, *30*–*35*). This synthetic polymer not only activates RLR pathways but also stimulates OAS/RNase L– and PKR-dependent defense mechanisms. Recently, we and others have shown that dsRNA generated by in vitro transcription using T7 RNA polymerase can be used to produce molecules with immunostimulatory potential similar to poly(I:C) (*35*–*37*). These dsRNA molecules have a triphosphate group at their 5′ ends, and it is widely accepted that the immunogenicity of dsRNA is due to the presence of this chemical group (*11*, *12*, *21*, *23*, *36*). Therefore, the following question arises: How does the presence of a cap at the 5′ ends of dsRNA modulate its immunogenic potential? In addition, mammalian transcripts have different versions of the cap structure that differ in the amount of 2′-*O*-methyl group attached to the 5′ end of RNA. On this basis, we can distinguish cap0, cap1, and cap2 (cap0 is unmethylated, cap1 has one 2′-*O*-methylation at the first transcribed nucleotide, and cap2 has two methyl groups at the first and second transcribed nucleotides). Recent studies on mammalian mRNAs revealed that 2′-*O*-methylations within the cap label transcripts as self-ones; thus, a cell treats triphosphorylated single-stranded RNAs (ppp-ssRNAs) or these ones with cap0 as a potential threat. RNA viruses in the course of evolution have acquired the ability not only to add a cap structure to their RNAs but also to generate their transcripts with a 2′-*O*-methylated version of the cap, i.e., transcripts with cap1. Does this mean that dsRNAs formed during the replication of such viruses avoid immune recognition inside human cells? Moreover, if 2′-*O*-methylation(s) reduce(s) the immunogenicity of the dsRNA, does this mean that only dsRNA with cap0 is immunogenic? A previous report showed that short blunt-ended dsRNA with cap0 bound to RIG-I with almost the same affinity as ppp-dsRNA (*20*); therefore, does this mean that dsRNA with cap0 triggers the innate immune response to the same extent as ppp-dsRNA? Furthermore, 2′-*O*-methyaltion of the first transcribed nucleotide within the cap structure has been shown to abrogate RIG-I binding to short dsRNA in vitro (*20*, *38*). Does this mean that the presence of only cap1 is sufficient for an RNA duplex to evade immune recognition in the cytoplasm and that the addition of a second 2′-*O*-methyl group within the cap structure (cap2) does not further reduce dsRNA immunogenicity? To answer these questions, we prepared a set of dsRNAs that differ in their 5′ end modifications. This was made possible by taking advantage of in vitro transcription reactions. By using appropriate cap analogs for RNA synthesis, we were able to generate ssRNAs with the cap structure of interest at their 5′ ends (*36*, *39*). To obtain dsRNA with a specific 5′ end modification, we prepared a pair of strands, sense and antisense, both with the same 5′ end modification, and annealed it (Fig. 1A). By using different cap analogs for the in vitro transcription, we obtained dsRNAs with a cap0, cap1, or cap2 structure at the 5′ end. Furthermore, our goal was to generate dsRNA of sufficient length to be recognized not only by RIG-I but also by host receptors whose mode of action depends primarily on the recognition of the RNA duplex. The optimal length to activate OAS proteins is more than 50 base pairs (bp) (*19*, *26*, *40*); for the PKR substrate, it should be at least 30 bp long (*15*). For MDA5, the dsRNA should be longer than 0.5 kilo–base pairs (kbp) (*23*), although other reports have shown that this host sensor is able to recognize much shorter transcripts (*24*, *41*). However, because the exact mechanism of dsRNA selection by MDA5, as well as its activation, has not been fully characterized, we decided to prepare a blunt-ended dsRNA 557 bp in length (Fig. 1B and fig. S1A). To ensure that the used transcripts were of high purity and free from T7 RNA polymerase by-products, such as dsRNA impurities and ssRNA molecules of unintended size, we performed high-performance liquid chromatography (HPLC) purification of the in vitro–transcribed RNA. Last, to obtain homogeneously capped transcripts, HPLC-purified RNAs were enzymatically treated to remove the uncapped versions. Once the sense and antisense strands used for dsRNA formation were purified, they were heated and cooled slowly to form duplexes. The efficiency of duplex formation was verified by agarose gel electrophoresis (Fig. 1C and fig. S1B).

With the set of 5′ end modified dsRNA in our hands, we tested whether the presence of the cap structure affects dsRNA immunogenicity. To this end, we transfected human A549 lung epithelial cells with dsRNA carrying cap1 or a triphosphate group at its 5′ end. After 5 hours, as we expected, the level of interferon-β (IFN-β) transcript was much higher in cells transfected with ppp-dsRNA than those in cells transfected with its counterpart bearing cap1 (fig. S2). This result encouraged us to perform genome-wide transcriptomic analysis to precisely define the contribution of each of 5′ end modifications to the immunogenicity of dsRNA. Analogically, RNA sequencing (RNA-seq) revealed that the most immunogenic version of the dsRNA was the one with a triphosphate group at the 5′ end, which was reflected in the highest number of up-regulated genes in all the samples analyzed (Fig. 1D and fig. S3). Gene Ontology (GO) analysis showed that the group of genes associated with the immune and antiviral pathways was the most up-regulated (Fig. 1E). The presence of the cap structure reduces the immunogenicity of dsRNAs. However, unexpectedly, the incorporation of 2′-*O*-methylation within the cap structure (cap1) almost completely abolished dsRNA immunogenicity (Fig. 1D and fig. S3). The presence of cap0, and therefore, the absence of 2′-*O*-methylation, results in a moderate immunostimulatory effect of dsRNA on A549 cells. In contrast, the addition of a second 2′-*O*-methylation at the 5′ end of the dsRNA (cap2) only slightly altered the gene expression profile observed for cap1-dsRNA–transfected cells, indicating that the presence of only one 2′-*O*-methylation is sufficient to severely reduce the immunogenic potential of the dsRNA (Fig. 1D and figs. S3 and S4). In addition, GO terms associated with innate immune or antiviral responses were no longer the most enriched in cells transfected with cap1- or cap2-dsRNA (Fig. 1E). The RNA-seq results were confirmed by independent analysis of the expression levels of selected ISGs (IFIT1, IFIT2, IFIT3, MDA5, OASL, and RIG-I) by reverse transcription quantitative polymerase chain reaction (RT-qPCR) (Fig. 1F). These results strongly suggest that the 5′ end of dsRNA is the primary trigger for cellular inflammation. The effect of the cap presence at the 5′ end of dsRNA on its immunogenicity is consistent with our knowledge about RIG-I activation: 2′-*O*-Methylation of the cap structure substantially reduces RIG-I affinity for dsRNA (*20*). Similarly, it is thought that 2′-*O*-methylation in the cap structure may also contribute to the evasion of dsRNA recognition by MDA5 (*12*, *25*), but other reports indicate that the length of the duplex in the transcript is the main activator of this receptor (*23*, *42*). Nevertheless, the dsRNA we prepared was able to evade recognition by the RLR pathways because of the 2′-*O*-methylation incorporated into its cap structure. Therefore, this tool allowed us to study the activation of cell growth inhibitory pathways independently of the cell’s inflammatory response.

### Capped dsRNAs are still detected by PKR

**Fig. 2.**
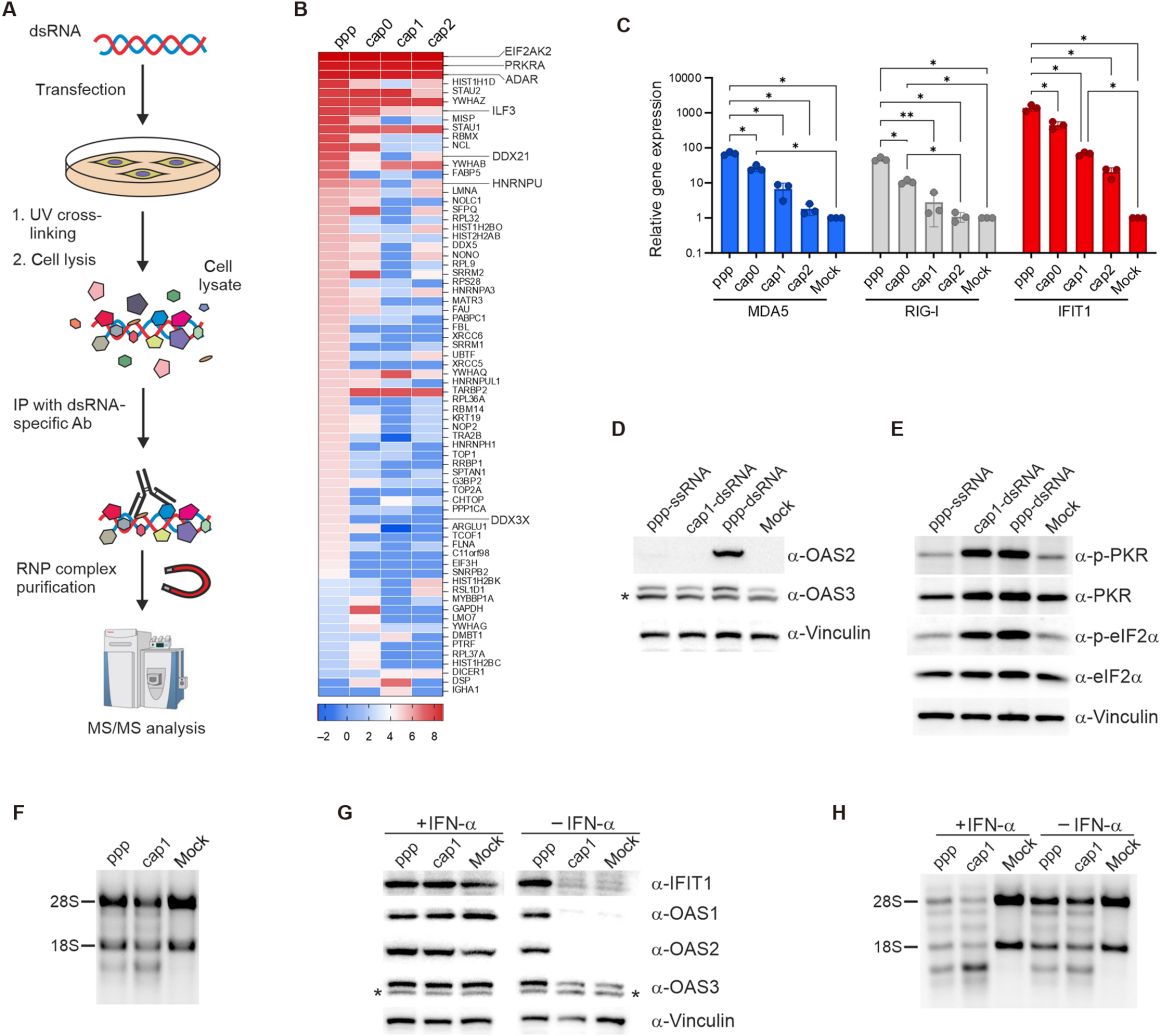
Less immunogenic dsRNA activates PKR and OAS/RNase L pathways. (**A**) Schematic of the proteomic approach: A549 cells are transfected with differently capped dsRNAs for 5 hours, UV cross-linked, and lysed, and dsRNA-containing RNP complexes are captured using dsRNA-specific antibodies. The protein composition is analyzed via MS/MS. (**B**) Protein specificity heatmap showing log_2_ intensity ratios of identified proteins across experimental conditions. Proteins with a specificity value >4.0 in at least one condition are displayed (full heatmap in fig. S6). (**C**) RT-qPCR analysis of RLR pathway activation in A549 cells transfected with dsRNAs bearing different 5′ end modifications for 5 hours. Data represent the means ± SD (*n* = 3). Statistical significance: *P < 0.05 and **P < 0.01 (one-way ANOVA with Tukey’s multiple comparisons test). Only statistically significant differences are shown. (**D** and **E**) ppp-ssRNA does not activate RLR or PKR pathways. (D) A549 cells are transfected with ppp-dsRNA, cap1-dsRNA, or ppp-ssRNA for 24 hours (ISG expression) or 7 hours (PKR and eIF2α phosphorylation by Western blotting). The asterisk (*) indicates a nonspecific band. (**F**) RNase L activity assessed by rRNA integrity. rRNA integrity was analyzed in A549 cells 24 hours posttransfection with ppp- or cap1-dsRNA using denaturing agarose gel. (**G**) RLR pathway activation with or without interferon (IFN-α, 200 U/ml) in A549 cells transfected with ppp- or cap1-dsRNA for 24 hours. The asterisk (*) indicates a nonspecific band. (**H**) RNase L activity depends on the dsRNA presence but is independent of the immune state. A549 cells are pretreated with IFN-α (200 U/ml) for 24 hours and transfected with dsRNA for 24 hours, and rRNA integrity is analyzed in denaturing agarose gel.

We hypothesized that the observations described above indicate one of two possibilities: Either dsRNAs with significantly reduced immunogenicity are no longer recognized by host receptors, or dsRNAs are still recognized by host receptors despite their lack of immunogenicity, and host cells can take countermeasures to eliminate the potential threat of dsRNAs. To answer these questions, we performed a dsRNA interactome capture experiment to determine whether modifications at the 5′ end of dsRNA affect their binding partners in living cells. Our goal was to identify proteins that are involved in dsRNA recognition as soon as they appear in the cytoplasm of the host cell and not proteins that are ISG products that play a role in cellular defense only after dsRNA has been successfully recognized by the cell’s innate immunity. Therefore, we first tested the duration of transfection to select appropriate conditions for studying the primary cellular response at the protein level. We verified that, 5 hours after transfection, there was no increase in the level of ISG products with highly immunogenic ppp-dsRNA, in contrast to 24 hours after transfection (fig. S5). Therefore, A549 cells were transfected with a set of dsRNAs with different 5′ end modifications for 5 hours. Moreover, to identify host proteins that bind to dsRNA as accurately as possible, we used cross-linked proteins and their associated nucleic acids by exposing plated cells to ultraviolet (UV) light. After cell lysis, only ribonucleoprotein (RNP) complexes formed on the dsRNA were fished out using dsRNA-specific antibodies. The captured RNP complexes were subjected to semiquantitative tandem mass spectrometry (MS/MS) (Fig. 2A). To ensure that the analyzed cells responded in the expected manner to differently capped dsRNAs, we tested the expression levels of three ISGs (IFIT1, MDA5, and RIG-I) in cell lysates (Fig. 2C). Proteomic analysis revealed that the more immunogenic the dsRNAs, the more proteins were bound to them (Fig. 2B and figs. S6 and S7). In addition, GO analysis showed that, among the proteins enriched in immunoprecipitates (IPs) are factors involved in the innate immune response, but their number decreased when 5′-capped dsRNA was delivered to the cells (fig. S7). However, regardless of the modification of the 5′ end of dsRNA, several proteins were always present in IPs, including factors involved in the innate immune response, namely adenosine deaminases acting on RNA (ADAR) and PKR (EIF2AK2) (Fig. 2B and figs. S6 and S7). ADAR is responsible for adenosine-to-inosine editing within endogenous transcripts in mammalian cells and plays a critical role in the regulation of innate immune activation by suppressing endogenous RNA sensing (*43*). However, ADAR activity on foreign dsRNA can have proviral effects (*44*, *45*). In addition, ADAR activity is unlikely to lead to global changes in host cell metabolism, which may help host cells rearrange their metabolism in response to viral threats. PKR, in contrast, is a dsRNA-binding kinase that, upon detection of viral threat, undergoes activation via autophosphorylation, which subsequently leads to the phosphorylation of the translation initiation factor eIF2α (*16*). PKR is one of the key sensors responsible for detecting foreign RNAs during viral infection (*2*, *9*). PKR activation leads to the shutdown of global host cell translation to minimize viral spread. Thus, PKR appears to be a protein that can recognize dsRNA regardless of its immunogenicity and adjust the cell’s metabolism to the emerging threat.

We then tested whether dsRNA with reduced immunogenicity is recognized by PKR and whether this cell receptor, upon detection of cap1-dsRNA, can trigger a signaling cascade leading to global translation repression. We also investigated whether the lack of cellular inflammatory activation affects the global shutdown of host protein biosynthesis by PKR. To this end, we examined the phosphorylation status of both PKR and eIF2α in cells transfected with the highly immunogenic ppp-dsRNA and a dsRNA with significantly reduced immunogenicity—cap1-dsRNA. First, we tested whether we could observe the activation of innate immunity because of the presence of immunogenic dsRNA. As expected, we observed substantially increased levels of ISG products (OAS2 and OAS3) in cells transfected with ppp-dsRNA but not with cap1-dsRNA for 24 hours (Fig. 2D). However, elevated levels of phosphorylated PKR (p-PKR) and phosphorylated eIF2α (p-eIF2α) were observed regardless of the dsRNA used for cell transfection (Fig. 2E). Furthermore, we were wondering whether PKR activation and subsequent phosphorylation of eIF2α occur whenever foreign RNA is present in the cell cytoplasm. Mayo and Cole suggested that PKR can bind not only to dsRNA but also to ssRNA, albeit with lower affinity (*46*). Nevertheless, recognition of ssRNA by PKR also leads to its activation in vitro. We therefore tested whether PKR activation is based solely on recognition of the structure of the RNA duplex or whether the presence of exogenous ssRNA is sufficient to lead to eIF2α phosphorylation. We have previously shown that transfection of mammalian cells with exogenous single-stranded transcripts, even those with triphosphate groups at their 5′ ends, did not lead to stimulation of the antiviral and interferon responses (*36*). Our Western blotting analysis showed exactly the same phenotype; we observed no increase in expression of ISG products upon transfection of A549 cells with ppp-ssRNA (Fig. 2D). Last, we showed that, when cells were transfected with ssRNA bearing a triphosphate group at its 5′ end, the levels of p-PKR and p-eIF2α remained unchanged compared to the mock control (Fig. 2E).

Together, we showed that the double-strandedness is an essential feature of exogenous transcripts for the activation of the PKR-dependent innate immune response pathway. Furthermore, this activation does not depend on either the immunogenic potential of the dsRNA or the activation of cellular inflammation.

### OAS/RNase L pathway functions independently of inflammation

The host cell adapts to potential dsRNA threats not only by slowing down global translation but also by eliminating ssRNAs that can be a source of dangerous dsRNAs, for example, during viral replication (*2*, *9*). The aforementioned ssRNA degradation is achieved primarily through activation of the OAS/RNase L pathway, in which OAS proteins sense dsRNAs and then produce 2′-5′-linked oligoadenylates (2-5As), which ultimately serve as activators of RNase L (*19*). Induction of this mechanism leads to degradation not only of the virus ssRNAs but also of the host molecules. Although OASs belong to the ISGs, i.e., their levels are greatly increased during viral infection, it has been suggested that the basal level of OAS proteins may be sufficient to activate RNase L even before the onset of inflammation (*32*). Thus, it appears that RNase L can be activated regardless of the immune state of the host cell but only if OASs produce enough 2-5As as activators of this endonuclease. Intriguingly, other reports have shown that, among the three OAS proteins, OAS3 has the highest affinity for dsRNA (*26*, *27*). In addition, RNase L activation has been demonstrated to depend primarily on OAS3 expression during viral infection (*27*).

Despite the fact that none of the oligoadenylate synthetases were identified in our proteomic analysis, the OAS3 protein seems to be a suitable candidate for a factor capable of responding effectively to dsRNA regardless of the immune state of the host cell. Our analysis of RLR pathway activation showed that the basal level of OAS3 was relatively high and moderately increased in A549 cells after interferon treatment (Fig. 2G). In contrast to OAS3, basal expression levels of OAS1 and OAS2 were limited. Considering this, we examined RNase L activity in A549 cells transfected with dsRNAs differing in immunogenic potential by checking the integrity of the 18S/28S ribosomal RNA (rRNA) isolated from these cells. We observed that 18S and 28S rRNAs were degraded regardless of the 5′ end modification (Fig. 2F), suggesting that activation of cellular inflammation is not necessary for OAS proteins to recognize dsRNAs and activate RNase L. Presumably, the observed phenomenon is caused by OAS3, because OAS1 and OAS2 levels are very low and unchanged in cells transfected with cap1-dsRNA (Fig. 2D). Last, as expected, treatment of A549 cells with IFN-α, although leading to a high up-regulation of all three OAS proteins (Fig. 2G), did not result in the appearance of rRNA fragmentation in mock-treated cells (Fig. 2H), meaning that only the presence of dsRNA in the cytoplasm of the cell activates the OAS/RNase L pathway. This activation is independent of the immune state of host cells.

### Epitranscriptomic marks do not substantially influence dsRNA immunogenicity

In addition to adding cap structures to their transcripts (*28*), viruses install epitranscriptomic marks within RNA to generate RNAs that resemble human transcripts as closely as possible (*29*). Internal modifications found in vRNA are the same as those present in the human transcriptome. Therefore, we decided to test whether the presence of any of the three most prevalent posttranscriptional modifications in the human RNA, i.e., m^6^A, Ψ, or m^5^C, could affect dsRNA immunogenicity (*29*). To study this, we prepared a set of dsRNAs by in vitro transcription in which each particular nucleotide was replaced with its modified version (fig. S8A). In other words, transcripts with m^6^A had all the adenosines in both dsRNA strands changed to their *N*6-methylated analogs. RNAs carrying Ψ and m^5^C were obtained in an analogous manner, i.e., in the sequence of the former transcripts, all uridines were replaced by Ψ’s, and for the latter, all cytidines were changed to their 5-methyl counterparts. In addition, dsRNAs with modifications were obtained in two versions, with triphosphate or cap1 at the 5′ ends of each dsRNA strand. Regardless of the chemical modifications introduced within the RNA sequence, in all cases, each capped transcript has an adenosine with a 2′-*O*-methyl group at the position of the first transcribed nucleotide. Therefore, we used a pppApG dinucleotide analog as the initiator of the in vitro transcription reaction to prepare triphosphorylated RNA containing internal m^6^A, which allowed us to obtain RNA starting with adenosine instead of m^6^A (fig. S8A). We verified the efficiency of modified dsRNA formation using an agarose gel (fig. S8B). In addition, the stability of RNA duplexes was tested using RNase I (fig. S8C), which specifically degrades single-stranded transcripts and preserves double-stranded molecules (*47*). The prepared dsRNAs were used to study the activation of the interferon pathway by introducing differently modified dsRNA into A549 cells, which transiently expressed the firefly luciferase gene under the control of the IFN-β promoter.

**Fig. 3.**
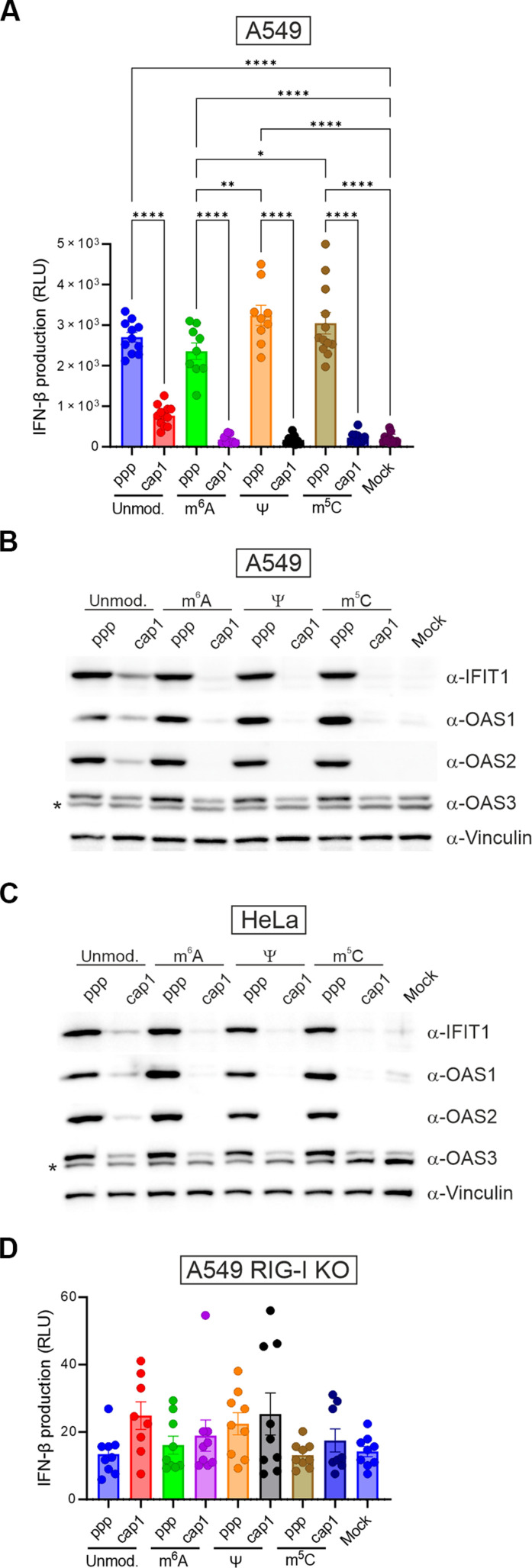
Epitranscriptomic marks do not substantially influence dsRNA immunogenicity. (**A**) IFN-β production in A549 cells assessed via luminescence of firefly luciferase under the control of the IFN-β promoter. Cells are transfected with dsRNA for 24 hours, and luciferase activity is measured. Bars represent the means ± SEM from at least three independent biological replicates (each with three technical replicates). Each point represents a technical replicate. Statistical significance: *P < 0.05, **P < 0.01, and ****P < 0.0001 (one-way ANOVA with Tukey’s multiple comparisons test). Only significant differences are shown. RLU, relative light units. (**B** and **C**) ISG product expression in A549 (B) or HeLa (C) cells transfected with ppp- or cap1-dsRNA carrying various epitranscriptomic marks for 24 hours, assessed by Western blotting. The asterisk (*) indicates a nonspecific band. (**D**) IFN-β production in A549 RIG-I KO cells assessed via luminescence of firefly luciferase as described in (A). No significant differences are observed (one-way ANOVA with Tukey’s multiple comparisons test). Verification of RIG-I KO in A549 RIG-I KO cells is shown in fig. S9.

We found that, regardless of the chemical modification introduced within ppp-dsRNA, it was immunogenic to the same extent as its unmodified counterpart (Fig. 3A). Modified cap1-dsRNAs were slightly less immunogenic than the unmodified capped variants; however, these differences were not statistically significant. Furthermore, at the protein level, we observed no differences in the expression levels of selected ISGs (IFIT1, OAS1, OAS2, and OAS3) between the unmodified ppp-dsRNAs and their modified counterparts (Fig. 3B). The presence of unmodified cap1-dsRNA in A549 cells resulted in a slight increase in ISG expression, whereas its modified counterparts did not lead to IFIT1 or OAS protein overexpression. This Western blot analysis aligns with IFN-β reporter assay, which indicated that, among capped dsRNA, only the unmodified variant exhibited some immunogenicity. These findings are further supported by RNA-seq (Fig. 1D and figs. S3 and S4) and RT-qPCR data (Fig. 1F), which also revealed slight immunogenicity for unmodified cap1-dsRNA.

In addition, to confirm that the presence of a triphosphate group is primarily responsible for dsRNA immunogenicity as a general phenomenon—and not a peculiarity specific to A549 cells—we examined ISG expression in another cell line, human cervical carcinoma HeLa cells, following transfection with the same set of modified and unmodified duplexes used previously. The results of the Western blot analysis were consistent with the data obtained for A549 cells (Fig. 3B): ISG (IFIT1, OAS1, OAS2, and OAS3) production was stimulated by the presence of a triphosphate group at the 5′ end of dsRNA regardless of the modified nucleotide incorporated into its sequence (Fig. 3C). Furthermore, the addition of cap1 at the 5′ end of dsRNA almost completely abolished the transcript’s immunogenicity.

Together, these results suggest that the primary feature of dsRNA that affects its immunogenic potential and leads to the activation of RLR pathways is the presence of the cap structure at transcript 5′ ends. The residual immunogenicity of cap1-dsRNA could be completely abolished by introduction of epitranscriptomic marks such as m^6^A, Ψ, and m^5^C.

Recognition of triphosphorylated blunt-ended dsRNAs as a potential threat to cellular homeostasis is primarily mediated by RIG-I (*11*, *13*, *20*–*22*). Nevertheless, the presence of longer duplexes, even those lacking a triphosphate group at the 5′ end, should also trigger an interferon response. MDA5 is thought to be the RNA sensor responsible for recognizing such duplexes and subsequently activating the immune response upon their detection (*23*, *24*). However, our data indicate that unmodified cap1-dsRNA is only slightly immunogenic (Fig. 3, A to C), which suggests that these transcripts may evade recognition by RIG-I and also by MDA5.

To better understand the contribution of each RLR sensor to dsRNA recognition, we examined the immunogenicity of our dsRNA set in A549 cells lacking RIG-I [A549 RIG-I knockout (KO)] using the previously mentioned luciferase IFN-β reporter assay. Unexpectedly, none of the tested dsRNAs induced an interferon response in cells lacking RIG-I (Fig. 3D). This finding suggests that, in human cells, RIG-I is primarily responsible for activating the immune response upon the presence of foreign RNA in the cytoplasm—at least under the conditions of our experimental setup.

### Epitranscriptomic marks do not prevent PKR recognition of dsRNA

**Fig. 4.**
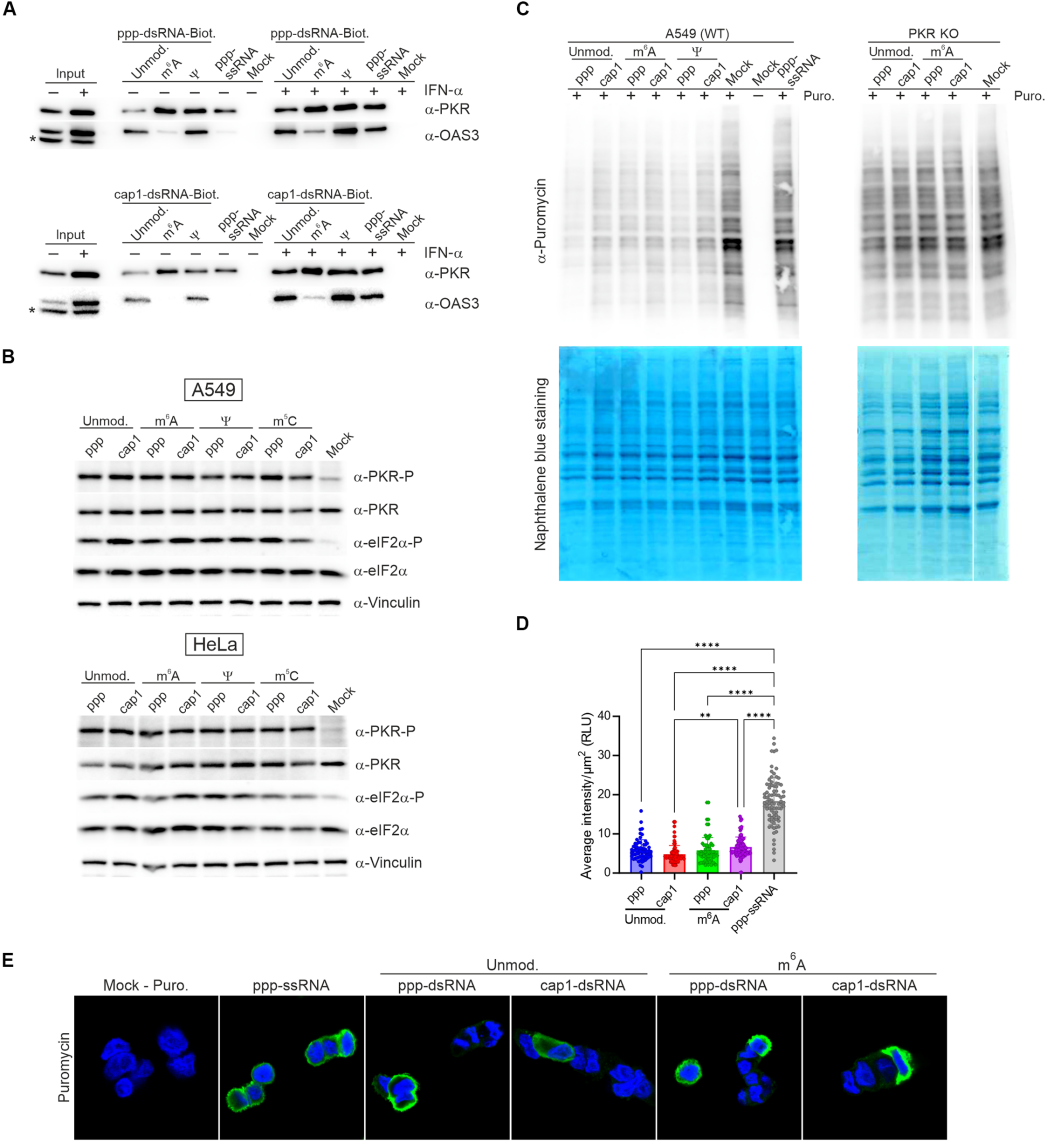
The presence of m^6^A, Ψ, and m^5^C within dsRNA does not affect its recognition by PKR. (**A**) Copurification of endogenous proteins from lysates of IFN-α–treated (200 U/ml) and untreated A549 cells with biotinylated ppp- or cap1-dsRNA. PKR and OAS3 are detected in precipitates by Western blotting. This is a cropped version of fig. S10. The asterisk (*) indicates an unspecific band. (**B**) Activation of the PKR pathway is not affected by the presence of posttranscriptional modifications within dsRNA. A549 or HeLa cells are transfected with ppp- or cap1-dsRNA carrying different epitranscriptomic marks for 7 hours, and the phosphorylation status of PKR and eIF2α is assessed by Western blotting. (**C** to **E**) The levels of newly synthetized proteins are assessed by puromycin incorporation assay. A549 and PKR KO cells are transfected with ppp- and cap1-dsRNA carrying different posttranscriptional modifications for 24 hours, and then puromycin (4 μg/ml) is added for 30 min. (C) The levels of newly synthetized proteins are assessed by Western blotting with anti-puromycin antibodies. Naphthalene blue staining is performed to control the loading. Verification of PKR KO cells is shown in fig. S11. [(D) and (E)] The levels of newly synthetized proteins are assessed by immunofluorescence with anti-puromycin antibodies. (D) Average intensity of puromycin staining in cells presented in (E) (between 81 and 173 cells are analyzed for each transfection). Bars represent the mean values ± SD from all analyzed cells. Each point represents data from one cell. Statistical significance: **P < 0.01 and ****P < 0.0001 (one-way ANOVA with Turkey’s multiple comparisons test). Only statistically significant differences were marked on the graph.

It is known that posttranscriptional modifications can affect the recognition of dsRNA by host factors that sense dsRNA on the basis of its double-strandedness and not on the modifications present at its 5′ ends (*2*, *9*, *29*). PKR is thought to be one of such sensors (*48*). It has been previously reported that tRNAs lacking posttranscriptional modifications can activate PKR in vitro and in cellulo (*49*, *50*). To investigate whether and how the three most abundant posttranscriptional modifications in the human transcriptome affect PKR binding to dsRNA, we performed pull-down experiments. We fished out proteins from the lysates of interferon-treated and untreated A549 cells using biotinylated dsRNA containing selected chemical modifications (Fig. 4A). Because all the tested posttranscriptional modifications affect the immunogenicity of dsRNA in the same way (Fig. 3, A to C), we used only two of them, m^6^A and Ψ, to prepare biotinylated dsRNA for the pull-down experiment. PKR binds to each dsRNA regardless of its modification. Similarly, the 5′ end cap did not affect dsRNA recognition by PKR. PKR is known to bind both dsRNA and ssRNA (*46*), and as expected, we found that it also bound to ssRNA in our analysis. These observations are consistent with the PKR and eIF2α phosphorylation status in A549 and HeLa cells studied after transfection with dsRNA carrying m^6^A or Ψ (Fig. 4B). None of the tested modifications affected phosphorylation of the inspected proteins.

We further investigated the hypothesis that posttranscriptional modifications do not prevent inhibition of global protein biosynthesis via a PKR-dependent pathway. We studied the levels of the newly synthesized puromycin-labeled proteins in A549 cells transfected with modified or unmodified dsRNAs (Fig. 4C). In Western blotting analysis, protein levels with incorporated puromycin were found to decrease in the presence of epitranscriptomic marks and 5′ end cap in dsRNAs. An analogous conclusion was drawn from the immunofluorescence studies of puromycin-labeled proteins in A549 cells (Fig. 4, D and E). Consistent with the assumption that PKR is the host factor primarily responsible for slowing the rate of protein biosynthesis in virus-infected cells, we observed that, regardless of the dsRNA used for transfection, no changes in translation were detected in PKR KO cells (Fig. 4C).

Together, obtained results lead to the conclusion that the presence of the three most widespread posttranscriptional modifications in the human transcriptome, i.e., m^6^A, Ψ, and m^5^C, within dsRNA does not affect dsRNA recognition by PKR and subsequent translational repression.

### *N*6-Methylation of adenosine shields dsRNA from OAS/RNase L

**Fig. 5.**
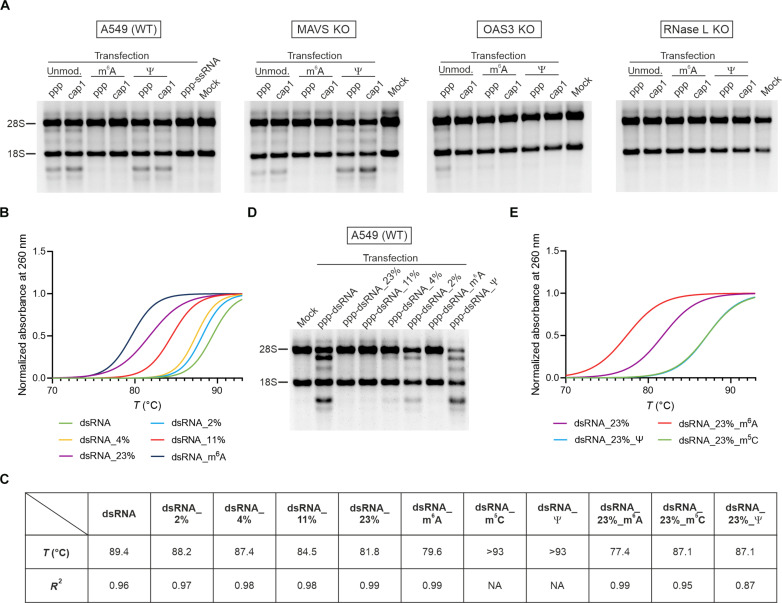
The presence of m^6^A within dsRNA influences RNase L activity. (**A**) RNase L activity assessed by rRNA integrity in A549 (wild type), MAVS KO, OAS3 KO, and RNase L KO cells. Total RNA is isolated 24 hours after transfection with posttranscriptionally modified (m^6^A or Ψ) ppp- or cap1-dsRNA and analyzed on denaturing agarose gel. KO validations for MAVS, OAS3, and RNase L are shown in fig. S11. (**B**) UV-VIS melting profiles of dsRNA bearing m^6^A or containing mismatches (2, 4, 11, and 23%). Fitted curves are shown; raw data are shown in fig. S13. Duplex formation efficiency for mismatched dsRNAs is shown in fig. S14A. (**C**) Comparison of melting temperatures (*T*_m_) of differently modified dsRNAs; *R*^2^ represents the goodness of fit of absorbance at 260 nm using the dose response-stimulation (four parameters) function. NA, not applicable. (**D**) RNase L activity assessed by rRNA integrity in A549 cells 2 hours after electroporation with ppp-dsRNA bearing m^6^A or Ψ or with ppp-dsRNA containing mismatches (2, 4, 11, and 23%). (**E**) UV-VIS melting profiles of dsRNA with epitranscriptomic marks (m^6^A, m^5^C, and Ψ) and 23% mismatches. The melting profiles of dsRNA_23%_m^5^C and dsRNA_23%_Ψ overlap. Fitted curves are shown; raw data are shown in fig. S13. Duplex formation efficiency for dsRNAs with mismatches and chemical modifications is shown in fig. S14B.

A pull-down experiment with biotinylated dsRNA showed that one of the tested epitranscriptomic marks (*N*6-methylation of adenosine) abolished OAS3 binding to dsRNA (Fig. 4A), in contrast to the unaffected interactions between RNA duplexes and PKR. As expected, this was observed independently of the 5′ end modification of the dsRNA. Whether the presence of m^6^A affects dsRNA recognition by other OAS proteins could not be confirmed in our experimental setup (fig. S10). However, the absence of OAS1 and OAS2 in IPs can be explained by the fact that these two proteins have a much lower affinity for dsRNA than OAS3 (*26*, *27*). To verify whether the in vitro observation that the presence of m^6^A affects OAS3 binding to dsRNA translates into a phenotype in living cells, we examined the integrity of rRNAs after transfection of A549 cells with various modified dsRNAs. We found that the presence of m^6^A protected dsRNA from recognition by OAS proteins in cellulo (Fig. 5A and fig. S12). The delivery of ppp-dsRNA containing m^6^A did not lead to the activation of RNase L in A549 cells. This observation supports the assumption that activation of cellular inflammation is dispensable for effective dsRNA recognition by OAS proteins, at least for OAS3 and activation of RNase L. Furthermore, in MAVS KO cells, i.e., cells with disabled RLR pathways, we observed degradation of rRNAs, unless these KO cells were transfected with dsRNA carrying the m^6^A modification (Fig. 5A). Only the elimination of RNase L completely inhibited rRNA degradation regardless of the dsRNA used. Similar to that, in RNase L KO cells, a significant decrease in rRNA degradation was observed in OAS3 KO cells. However, some rRNA degradation can still be observed in OAS3 KO cells upon delivery of immunogenic ppp-dsRNA, but this is probably due to the increased expression of OAS1 and OAS2 and activation of RLR pathways. Notably, this rRNA degradation is visible only when OAS3 KO cells are transfected with unmodified ppp-dsRNA. No signs of rRNA degradation are observed upon transfection with transcripts bearing Ψ. This difference could be due to Ψ affecting dsRNA binding with OAS1/2, although this requires further confirmation. However, in the case of ppp-dsRNA with m^6^A, no rRNA degradation was observed either in wild type or in OAS3 KO cells, strongly suggesting that this chemical modification protected dsRNA not only from recognition by OAS3 but also from detection by OAS1 and OAS2.

It is well established that the introduction of modified nucleotides, in place of their unmodified counterparts, can influence RNA structural stability. m^6^A, in contrast to m^5^C and Ψ, has been shown to destabilize RNA structures (*51*–*54*). Therefore, we sought to investigate how the presence of each of these modifications affected the stability of our dsRNA. To address this question, we spectrophotometrically measured the melting temperature (*T*_m_) of RNA duplexes (Fig. 5, B and C, and fig. S13). This analysis revealed that the introduction of m^6^A into dsRNA significantly affects RNA thermal stability, reducing the melting temperature from 89.4° to 79.6°C. This decrease in duplex stability is comparable to that of a dsRNA with 23% randomly introduced mismatches, which was prepared by substituting all adenines with guanines in one strand of the duplex (*T*_m_ of dsRNA_23% is 81.8°C). As expected, the number of mismatches introduced into the dsRNA negatively correlates with its melting temperature. The introduction of just 4% mismatches—which only slightly affects thermal stability—almost completely blocks activation of the OAS/RNase L pathway (Fig. 5D). In contrast, the presence of m^5^C or Ψ has an opposing effect on dsRNA thermal stability. However, we were unable to precisely determine the melting temperatures of dsRNA containing these modifications because of limitations in our experimental setup (fig. S13). Specifically, we were unable to capture UV absorbance from single-stranded transcripts at temperatures below 93°C. Therefore, to verify unambiguously that the presence of m^5^C or Ψ stabilizes RNA duplexes, we prepared an additional set of dsRNAs. Each dsRNA in the new set has a triphosphate group at its 5′ end. Knowing that substituting all adenines with guanines in the sense strand (dsRNA_23%) significantly decreases duplex stability, we tested whether the introduction of m^5^C or Ψ in the antisense strand would increase thermal stability and whether the incorporation of m^6^A would further destabilize the duplex. UV measurements corroborated our hypothesis: The presence of m^5^C or Ψ significantly increased the thermal stability of dsRNA_23% (*T*_m_ for both dsRNA_23%_m^5^C and dsRNA_23%_Ψ was 87.1°C), while the incorporation of m^6^A decreased the *T*_m_ to 77.4°C (Fig. 5, C and E). On the basis of this, we conclude that the presence of m^6^A within an RNA duplex affects the OAS/RNase L pathway by relaxing the RNA duplex structure, which specifically hinders dsRNA recognition by OAS proteins.

### PKR clusters on dsRNAs regardless of the immune state of the cell

**Fig. 6.**
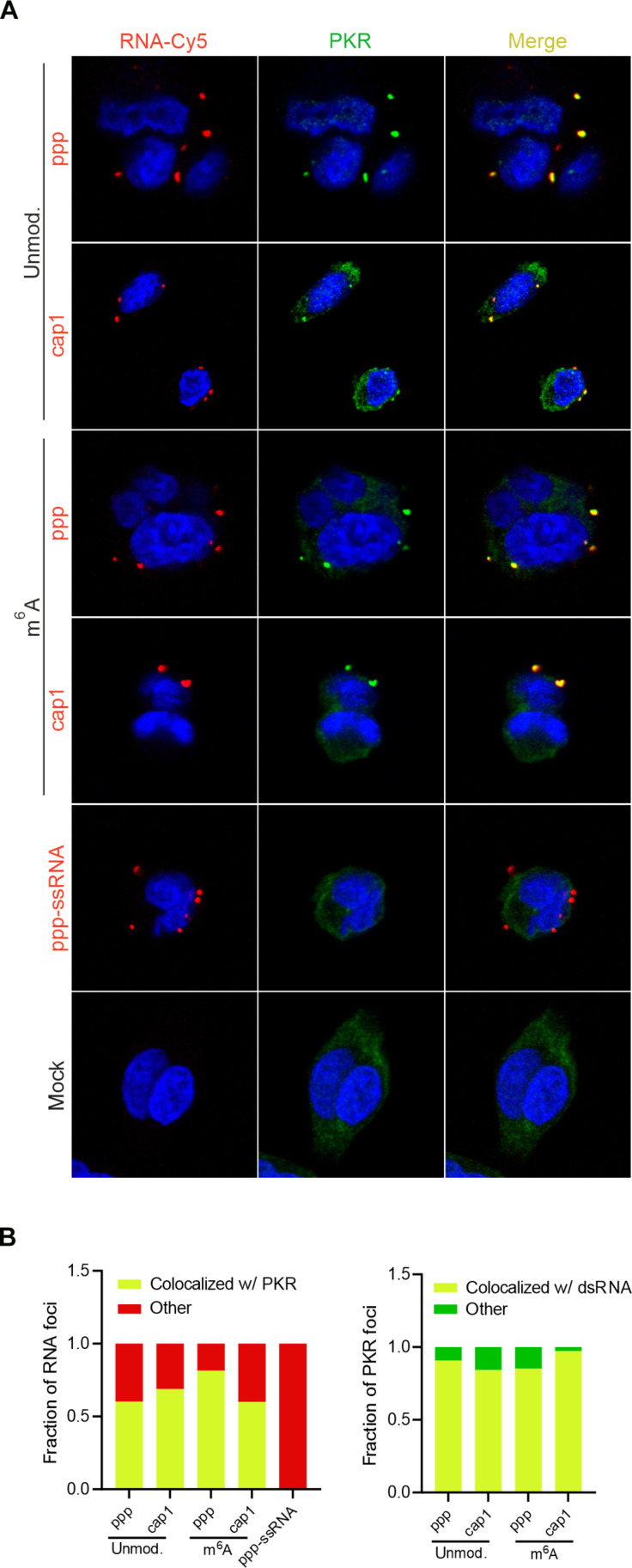
PKR forms clusters on differently modified dsRNA. (**A**) Immunofluorescence analysis of PKR and Cy5-labeled RNA in A549 cells. Cells are transfected with Cy5-labeled ppp- or cap1-dsRNA (with or without m^6^A) or Cy5-labeled ppp-ssRNA. (**B**) Quantification of (left) Cy5-labeled RNA colocalization with PKR and (right) PKR colocalization with Cy5-labeled RNA. Bars represent all foci counted across 127 to 874 cells per transfection. Yellow indicates RNA-PKR–colocalized foci, while red and green indicate foci with only PKR or RNA signals, respectively.

First, to repress translation, PKR is activated through autophosphorylation upon binding to dsRNAs and subsequently undergoes dimerization (*55*–*58*). Only as a dimer, PKR is capable of phosphorylating eIF2α, which subsequently leads to translational shutdown (*59*, *60*). In addition, Zappa *et al.* (*61*) recently showed that PKR in human cells transfected with poly(I:C) forms not only dimers but also higher-order assemblies. The exact reason why PKR forms clusters is not yet clear, but it has been proposed that PKR clustering may fine tune its activity; translational inhibition is moderately reduced by PKR clustering (*61*). Disruption of PKR clusters in cellulo leads to a moderate increase in eIF2α phosphorylation. Because we already knew that posttranscriptionally modified dsRNA could be effectively recognized by PKR (Fig. 4, A to E), we wondered whether the presence of epitranscriptomic marks could fine tune PKR activity through clustering. Furthermore, we were interested in whether the immunogenic potential of dsRNA, and thus, the immune state of the host cells, could influence this phenomenon. We prepared a set of Cy5-labeled triphosphorylated and capped versions of dsRNAs. Because none of the chemical nucleotide modifications tested affected PKR binding or translation repression, we examined the clustering phenomenon only for dsRNAs with m^6^A and their unmodified counterparts. Upon transfection of fluorescent dsRNAs into A549 cells, we observed spots in the cell cytoplasm containing introduced transcripts (Fig. 6A), as previously reported (*30*, *35*, *61*). In addition, we found that most foci with Cy5-labeled dsRNA (from 60 to 81%) colocalized with PKR, regardless of the modification at the 5′ end of the transcript (Fig. 6B). The colocalization of PKR with dsRNA was also not affected by the presence of m^6^A in dsRNA. Almost all signals recorded for PKR (from 84 to 97%) overlapped with those observed for dsRNA. The higher percentage of PKR colocalized with dsRNA than dsRNA colocalized with PKR suggests that dsRNA first forms cytoplasmic foci, and then PKR is attracted to these foci and begins to form clusters. This hypothesis is supported by the fact that PKR is not required for the formation of dsRNA condensates. The dsRNA foci were still visible in PKR KO cells transfected with Cy5-labeled dsRNA (fig. S15, A and B). Our observation is consistent with that of a recent study by Corbet *et al.* (*30*) showing that the presence of PKR is dispensable for the formation of poly(I:C) foci.

RNA condensates were also present in cells transfected with ssRNA (Fig. 6A). However, this observation is expected, as the occurrence of punctate signal containing mRNAs in mammalian cells transfected by lipofection is already well documented (*62*–*67*). The aim of PKR clustering is to limit its activity (*61*), namely to reduce eIF2α phosphorylation, and it is possible that also foci containing ssRNA may form clusters with PKR. However, in this case, PKR did not colocalize with the RNA condensates (Fig. 6, A and B). Thus, the lack of colocalization of PKR with ssRNA provides further evidence that, despite its affinity for ssRNA in vitro (*46*), this kinase specifically recognizes only dsRNA in living cells. Furthermore, the observation that both ssRNA and dsRNA form RNA condensates indicates that the presence of these foci is not due to the presence of specific nucleic acids in the cell cytoplasm. In all these cases, the transcripts were introduced into cells by means of lipofection, which is based on the formation of complexes, i.e., lipoplexes, composed of nucleic acids and positively charged lipids present in the transfection reagent (*62*, *68*). Thus, the spots observed in the cells are only lipoplexes with the cargo (nucleic acids) not released yet (*68*–*70*). This clearly demonstrates that the formation of RNA condensate is independent of PKR action, and this phenomenon probably occurs independently of the action of any other host cell protein. Moreover, the presence of RNA condensates formed by foreign transcripts in the cell is simply due to the way nucleic acids are introduced into the cell.

## DISCUSSION

Our studies have shown that effective dsRNA recognition in mammalian cells is achieved independent of RLR pathway activation. This process is mediated by the action of host cell sensors involved in cell growth inhibition such as OASs and PKR. Although they are ISGs, their basal levels are sufficiently high to mount action against dsRNA without prior induction of inflammation. As we did not observe an increase in the expression of ISG products in cells with activated OAS/RNase L and PKR pathways, we postulate that the activation of these defense mechanisms does not contribute to cellular inflammatory processes or that this contribution is low. Activation of cellular inflammation was observed for dsRNA with a triphosphate group at the 5′ ends and, to some extent, in cells treated with dsRNA carrying a cap0 structure. In contrast, the presence of a 2′-*O*-methylated version of the cap structure almost completely abolished dsRNA immunogenicity. We observed only a slight up-regulation of some gene expression in cells treated with cap1- or cap2-dsRNA. The combined 2′-*O*-methylated cap structure and epitranscriptomic marks completely abolished dsRNA immunogenicity.

Among the RLRs, two of them, namely RIG-I and MDA5, are directly involved in dsRNA recognition and activation of the signaling cascade leading to the expression of ISGs, cytokines, and interferons (*11*–*13*, *21*). It is well documented that RIG-I recognizes dsRNA on the basis of its 5′ end structure and has the highest affinity for triphosphorylated blunt-ended dsRNA, but only the presence of a 2′-*O*-methyl group within the cap structure eliminates RIG-I’s interaction with dsRNA (*2*, *11*, *20*–*22*, *38*). This is consistent with our observations that only the presence of a 2′-*O*-methylated cap structure significantly reduces the immunogenic potential of dsRNA. Our data showed that transfection of cells with dsRNA containing only cap0 resulted in a notable decrease in ISG expression. The almost complete lack of immunogenicity of cap1- and cap2-dsRNAs raises questions about the role of MDA5 in dsRNA detection. MDA5, unlike RIG-I, is not likely to recognize dsRNA on the basis of modifications of its 5′ ends but is rather activated by the long duplex structure (*23*, *24*). It is thought that the optimal MDA5 substrate should be longer than 0.5 kbp (*23*), but there are also reports that MDA5 is able to recognize much shorter dsRNAs than 0.5 kbp (*24*, *41*). Some studies claim that the presence of a 5′ end modification also affects MDA5’s dsRNA recognition in the same way as RIG-I (*12*, *25*). However, our current studies showed the lack of cell stimulation with dsRNA regardless of its 5′ end modifications upon the absence of RIG-I. Therefore, there are two possible scenarios to explain why our prepared dsRNA avoids recognition by MDA5: (i) The basal level of MDA5 is too low to produce an effective response against dsRNA without an increase in its expression as what happens during inflammation, or (ii) it is simply too short; we prepared a duplex of 557 bp in length, and MDA5 only senses much longer substrates. Determining the exact answer to this question is important for fully understanding the exact mechanism of MDA5 activation; however, this is beyond the scope of the current study. Nevertheless, the observation that the cap structure almost completely reduced dsRNA immunogenicity is consistent with viral evasion strategies. Almost all RNA viruses have evolved the ability to add a cap structure to their RNAs (*28*). This takes advantage of cap-dependent translation, the main process of protein biosynthesis in mammalian cells, but also prevents immune recognition.

Knowing that we can disable RLR pathways by installing a 2′-*O*-methylated cap structure on dsRNA, we have an ideal tool to study the response of cell growth inhibitory pathways to dsRNA independent of cellular inflammation. Using this tool, we separated the actions of the OAS/RNase L and PKR pathways from that of the RLR-based cellular response. Both growth-inhibitory pathways responded effectively to dsRNAs even in the absence of cellular inflammation. OAS3 recognizes dsRNA and activates RNase L, leading to ssRNA degradation. Following dsRNA recognition, PKR phosphorylates eIF2α and represses global translation. The degree of activation of both pathways was the same in cells transfected with nonimmunogenic and immunogenic dsRNAs. This means that even if dsRNAs can evade recognition by the RLRs during viral infection, the host cell is not powerless and is still able to take appropriate countermeasures to eliminate this threat. However, this observation raises the following question: What is the benefit of activating RNase L and PKR without inducing cellular inflammation? Alternatively, is such a situation undesirable and does it give the cell no advantage in fighting potential threats? When analyzing our data, it is important to remember that dsRNAs are not only of viral origin but can also be endogenously derived. The higher abundance of dsRNAs in the cytoplasm of the cell may also be related to more frequent retrotransposition events, higher expression of long noncoding RNAs, or the presence in the cytoplasm of more transcripts with long 3′ untranslated regions that are prone to duplex formation (*1*). The question then arises as to whether it is necessary in such situations to induce inflammation and to warn the neighboring cells with cytokines and interferons that there is a serious threat to the homeostasis of the tissue or of the organism. Alternatively, it may be better to slow down cellular metabolism and take appropriate countermeasures to eliminate inappropriate levels of potentially dangerous transcripts; if this does not work, RNase L and PKR activity may eventually lead to cell apoptosis (*71*–*73*). If a mammalian cell chooses the latter scenario and tries to cope with dsRNA without involving other cells present in the tissue, the problem could be solved at the single-cell level without wasting the energy of the entire organism to deal with inflammation. Thus, the basal activity of OAS3 and PKR, which is maintained in cells growing under optimal conditions (*32*), appears to be an evolutionary adaptation to eliminate inappropriate levels of endogenously derived dsRNA, which poses no potential threat to neighboring cells in the tissue. Unfortunately, viruses have evolved a highly effective strategy to protect their duplexes from the primary cellular response of the host, which is particularly important for RNA viruses that replicate in the cytoplasm. Without a special shielding mechanism, viral dsRNA replication intermediates are fully exposed to host receptors, which prevents viruses from replicating efficiently. The strategy that probably all RNA viruses have developed is to restrict their replication to special factories called replication organelles (*74*). Replication organelles are membrane organelles that isolate the site of viral replication from the host’s innate immunity, giving viruses a double protection, hiding replication intermediates that are not yet capped, and protecting the double-strandedness itself generated during replication.

Another strategy used by viruses to evade recognition of their transcripts by the host’s innate immunity is to introduce posttranscriptional modifications similar to those present in host RNA (*2*, *9*, *29*). Although the presence of these modifications in vRNA was first characterized approximately half a century ago (*75*, *76*), the extent to which viruses benefit from their presence in their transcripts remains unknown. It is also possible that the presence of posttranscriptional modifications in vRNA may be a side effect of viral replication in mammalian cells. Because all mammalian transcripts contain modified nucleotides, it is likely that viral transcripts would undergo the same posttranscriptional modifications as host RNA. Therefore, we tested how the three most common epitranscriptomic modifications in the human transcriptome, i.e., Ψ, m^6^A, and m^5^C, affect dsRNA-host cell interactions. Using in vitro transcription, dsRNAs carrying each of the three modified nucleotides were prepared. Unexpectedly, our data showed that the presence of any of the tested modifications did not significantly affect the dsRNA immunogenicity. However, it is worth mentioning that the immunogenicity of dsRNA was completely abrogated when the installation of a cap1 structure at the 5′ end was combined with the presence of epitranscriptomic marks. Nevertheless, this observation supports the notion that the 5′ end modification is a key player in the regulation of cellular inflammation in response to dsRNA. This appears to be the case at least in cells that rely on cytoplasmic dsRNA sensing. However, its effect on phagocytic cells, such as macrophages or dendritic cells (DCs), which recognize dsRNA not only through RLRs but also through Toll-like receptors (*2*, *9*, *77*), has not been determined. Some clues come from our recent studies in which we transfected murine DCs (mDCs) with crude in vitro–transcribed mRNA, i.e., mRNA containing double-stranded impurities generated by T7 RNA polymerase. We found that, in mDCs, crude triphosphorylated in vitro–transcribed mRNA was more immunogenic than its cap1 version (*36*).

Furthermore, using dsRNAs with a 2′-*O*-methylated cap structure and carrying posttranscriptional modifications, we examined the effects of Ψ, m^6^A, and m^5^C on cell growth inhibitory pathways. Despite previous suggestions that PKR activity may be regulated by posttranscriptional modifications (*48*–*50*), we observed no effect of any of these modifications on PKR-induced translational repression. As PKR forms higher-order assemblies in addition to dimers in cellulo (*61*), we also showed that none of the tested modifications affected PKR clustering on dsRNAs. Similarly, the immunogenic potential of dsRNA did not prevent PKR from forming higher-order assemblies. However, our immunofluorescence analysis showed that the presence of dsRNA foci in transfected mammalian cells is a side effect of lipofection rather than a unique ability of dsRNA to form condensates inside living cells. Analogous foci were observed when ssRNA was delivered into A549 cells via lipofection.

Last, we found that the presence of one of the posttranscriptional modifications tested, m^6^A, completely abolished dsRNA binding to OAS3 and thus did not induce RNase L–mediated ssRNA degradation. Nevertheless, we can extrapolate this observation to the other two OAS proteins, especially as it is OAS3 that has the highest affinity for dsRNA among the OAS proteins (*26*, *27*). Furthermore, after transfection of OAS3 KO cells with unmodified and m^6^A-modified dsRNAs, only the former induced slight rRNA degradation in this KO cell line. This suggests that OAS1/2 binding to dsRNA is also abolished by the presence of m^6^A.

It is widely accepted that the presence of posttranscriptional modifications within a transcript can remodel its structure, with m^6^A destabilizing the RNA duplex, while the presence of m^5^C or Ψ has the opposite effect (*51*–*54*). To test whether the relaxation of the duplex structure caused by m^6^A is responsible for the abrogation of dsRNA-OAS interactions, we performed UV-visible (UV-VIS) measurements of dsRNA thermal stability. These measurements revealed that only the incorporation of m^6^A decreases the dsRNA melting temperature, while the presence of the other two epitranscriptomic marks, Ψ and m^5^C, stabilizes the RNA duplex. This decrease in dsRNA thermal stability is likely caused by imperfect base pairing between the dsRNA strands, as the *N*6-methyl group on adenine interferes with its binding to uridine (*78*). The presence of mismatched dsRNA in the cytoplasm does not lead to RNase L activation. Moreover, since m^6^A affects dsRNA binding by OAS proteins but not by PKR, this observation suggests a novel level of regulation in dsRNA recognition by host receptors, potentially based on the complementarity of the duplex.

Using in vitro–transcribed dsRNA, we have demonstrated that the key feature of dsRNA that regulates the onset of cellular inflammation in response to its recognition as a potential viral threat is its modification at the 5′ end. We showed that the presence of the cap0 structure alone notably reduced the immunogenic potential of dsRNA. Furthermore, the introduction of dsRNA carrying a cap structure with only one 2′-*O*-methylation into mammalian cells almost completely abolishes the onset of cellular inflammation. Nevertheless, mammalian cells can effectively respond to the potential threat of dsRNA, even without activating inflammatory pathways, through the action of OAS/RNase L– and PKR-dependent mechanisms. The answer to the question of why either of these two growth inhibitory pathways is fully functional in the absence of inflammation is that, in addition to coping with the viral threat, appropriate countermeasures should be taken when abnormal levels of endogenously derived dsRNA appear in the cytoplasm of the cell. In such situations, the expression of cytokines and interferons appears to be an unnecessary expenditure for the organism.

## MATERIALS AND METHODS

### Cell lines

A549 (CCL-185) and HeLa (CCL-2) cell lines from American Type Culture Collection, A549 RIG-I KO generated in this study, and A549 MAVS KO, A549OAS3 KO, A549 PKR KO, A549 RNase L KO cell lines provided by S. Weiss (*27*, *79*) were maintained at 5% CO_2_ and 37°C in Dulbecco’s modified Eagle’ medium (Gibco, 21969035) supplemented with heat-inactivated fetal bovine serum (10%, v/v) (Sigma-Aldrich, F9665), GlutaMAX (1%, v/v) (Gibco, 35050061), and penicillin/streptomycin (1%, v/v) (Gibco, P4333).

### Plasmids

To generate plasmid encoding either a sense or antisense strand for RNA duplex formation, part of the *Gaussia* luciferase coding sequence was amplified via PCR using the pJET_Gluc_A128 (*36*) plasmid as a template, primers (table S1), and Phusion High-Fidelity DNA Polymerase (Thermo Fisher Scientific, F530). The PCR amplicons were ligated into the pJET1.2 plasmid using CloneJET PCR Cloning Kit (Thermo Fisher Scientific, K1231). Subsequently, pJET1.2 plasmids with a cassette encoding either the sense or antisense RNA strand were digested with Ade I (Thermo Fisher Scientific, FD1234) and Xba I (Thermo Fisher Scientific, FD0685) and each insert was ligated into the Ade I/Xba I–treated pJET_Gluc_A128 with the use of T4 DNA ligase (Thermo Fisher Scientific, EL0013), giving rise to the pJET_Gluc1 and pJET_Gluc2 constructs encoding sense and antisense strands of dsRNA, respectively.

Sequences encoding an antisense strand of the RNA duplex, enabling generation of dsRNA with randomly distributed mismatches, were synthetized by Thermo Fisher Scientific GeneArt Service. These sequences were encompassed by restriction sites for Ade I and Xba I, allowing them to be incorporated into the Ade I/Xba I–treated pJET_Gluc1 plasmid, giving rise to pJET_Gluc1_23%, pJET_Gluc1_11%, pJET_Gluc1_4%, and pJET_Gluc1_2%.

The pSpCas9(BB)-2A-Puro (PX459) V2.0 plasmid [gift from F. Zhang, Addgene plasmid no. 62988 (*80*)] was used to generate the RIG-I KO cell line. Overlapping oligonucleotides (table S1) were annealed in T4 DNA ligase buffer and ligated into the Bpi I sites in pSpCas9(BB)-2A-Puro (PX459) V2.0 using T4 DNA ligase, giving rise to three pSpCas9(BB)-2A-Puro-RIG-I plasmids.

### Antibodies

Rabbit anti-eIF2α (Cell Signaling, 9722S, RRID:AB_2230924), rabbit anti-phospho-eIF2α (Ser51) (Cell Signaling, 9721S, RRID:AB_330951), rabbit anti-GAPDH (Novus, NB300-327, RRID:AB_10001915), rabbit anti-IFIT1 (Invitrogen, PA3-848, RRID:AB_1958733), rabbit anti-MAVS (Invitrogen, PA5-17256, RRID:AB_10979584), rabbit anti-OAS1 (Invitrogen, PA5-82113, RRID:AB_2789274), mouse anti-OAS2 (Invitrogen, MA5-26552, RRID:AB_2724898), rabbit anti-OAS3 (Invitrogen, PA5-59494, RRID:AB_2644932), rabbit anti-PKR (Cell Signaling, 12297S, RRID:AB_2665515), mouse anti-Puromycin (Sigma-Aldrich, MABE343, RRID:AB_2566826), rabbit anti-RIG-I (Cell Signaling, 3743S, RRID:AB_2269233), rabbit anti-RNase L (Cell Signaling, 27281S, RRID:AB_2798941), and rabbit anti-Vinculin (Invitrogen, 700062, RRID:AB_2532280) were used at 1:1000, whereas Goat anti-Mouse IgG (H + L) Secondary Antibody HRP (Invitrogen, 62-6520, RRID:AB_2533947) and Goat anti-Rabbit IgG (H + L) Secondary Antibody HRP (Invitrogen 65-6120, RRID:AB_2533967) were used at 1:10000 for Western blotting. Rabbit anti-PKR and mouse anti-Puromycin were used at 1:250, whereas Alexa Fluor 488-AffiniPure Donkey Anti-Mouse IgG (H + L) (Jackson ImmunoResearch, 715-545-150, RRID:AB_2340846) and Donkey anti-Rabbit IgG (H + L) Highly Cross-Adsorbed Secondary Antibody Alexa Fluor 488 (Invitrogen, A-21206, RRID:AB_2535792) were used at 1:1000 for immunofluorescence.

### Generation of the RIG-I KO cell line

A549 cells were cultured to 80% confluency in a six-well plate and transfected with an equimolar mixture of three pSpCas9(BB)-2A-Puro-RIG-I plasmids (600 ng in total) using Lipofectamine 3000 (Invitrogen L3000001). Forty-eight hours posttransfection, the culture medium was replaced with fresh medium containing puromycin (1.5 μg/ml) (InvivoGen ant-pr-1). After 3-day selection, the medium was replaced for an antibiotic-free one. When the cells reached 80% confluency, they were serially diluted to obtain single-cell clones in each well of a 96-well plate. Individual colonies were then propagated and screened for RIG-I expression via Western blotting.

### In vitro transcription

RNA used in this study (table S2) was obtained as described previously (*36*, *39*) with some modifications. A pJET-based construct linearized with PacQI (New England Biolabs, R0745) restrictase served as a template for in vitro transcription. The reaction mixture contained RNA Pol buffer [40 mM tris-HCl (pH 7.9), 10 mM MgCl_2_, 1 mM dithiothreitol (DTT), and 2 mM spermidine], DNA template (40 ng/μl), and a mixture of nucleotides:

1) Cytidine 5′-triphosphate (CTP), guanosine 5′-triphosphate (GTP), and uridine 5′-triphosphate (UTP) (2 mM each) and 0.5 mM adenosine 5′-triphosphate (ATP) and 1.5 mM appropriate cap analog for capped RNA [m^7^GpppApG, m^7^GpppA_m_pG, or m^7^GpppA_m_pG_m_pG for RNA capping with cap0, cap1, or cap2, respectively (*36*, *39*)] or 2 mM ATP (no cap analog) for noncapped triphosphorylated transcript to obtain unmodified transcripts.

2) CTP, GTP, and pseudo-UTP (Jena Bioscience NU-1139S; 2 mM each) and 0.5 mM ATP and 1.5 mM appropriate cap analog for capped RNA (m^7^GpppA_m_pG) or 2 mM ATP (no cap analog) for noncapped triphosphorylated transcript to obtain transcripts with pseudo-UTP.

3) 5-Methyl-CTP (Jena Bioscience NU-1138L), GTP, and UTP (2 mM each) and 0.5 mM ATP and 1.5 mM appropriate cap analog (m^7^GpppA_m_pG) for capped RNA or 2 mM ATP (no cap analog) for noncapped triphosphorylated transcript to obtain transcripts with 5-methyl-CTP.

4) CTP, GTP, and UTP (2 mM each) and 0.5 mM *N*6-methyl-ATP (Jena Bioscience NU-1101L) and 1.5 mM appropriate cap analog (m^7^GpppA_m_pG) for capped RNA or 0.5 mM *N*6-methyl-ATP and 1.5 mM pppApG dinucleotide (Biolog T050) as a transcription initiator for noncapped triphosphorylated transcript to obtain transcripts with *N*6-methyl-ATP; RiboLock RNase Inhibitor (1 U/μl; Thermo Fisher Scientific, EO0382), homemade T7 RNA polymerase, and pyrophosphatase (1 U/μl; Thermo Fisher Scientific, EF0221).

The mixture was incubated at 37°C, and after 2 hours, deoxyribonuclease I (DNase I) (Thermo Fisher Scientific, EN0521) was added (0.1 U/μl) for an additional 30 min. The crude RNA was purified using the Monarch RNA Cleanup Kit (New England Biolabs, T2040). Next, RNA was purified using an Agilent 1260 Infinity HPLC system and XBridge Premier Oligonucleotide BEH C18 Column, 130 Å, 2.5 μm, 4.6 mm by 150 mm (Waters, 186009903). HPLC was conducted at 55°C, with a linear gradient of buffer B [0.1 M triethylammonium acetate (pH 7.0) and 50% acetonitrile] from 21.9 to 26.5% (for unmodified RNA and transcripts with pseudo-UTP), 22.5 to 27.1% (for transcripts with 5-methyl-CTP), or 24.0 to 28.6% (for transcripts with *N*6-methyl-ATP) in buffer A [0.1 M triethylammonium acetate (pH 7.0)] over 30 min and at a flow rate of 1 ml/min. RNAs from the collected fraction were recovered by isopropanol precipitation. Noncapped RNAs from samples with capped RNAs were removed by two separate treatments, i.e., with RNA 5′ polyphosphatase (Biosearch Technologies, RP8092H) and XRN-1 (New England Biolabs, M0338), including purification using a Monarch RNA Cleanup Kit step in between. Transcripts after enzymatic reactions were purified again using the Monarch RNA Cleanup Kit.

### RNA labeling

Biotinylation or labeling with Cyanine-5 was performed with poly(A) polymerase (PAP) as described previously (*81*). An HPLC-purified antisense RNA strand (10 μM) and 0.1 mM 2′-azido-2′-dATP nucleotide analog (Jena Bioscience NU-976S) were incubated at 20 μl with RiboLock RNase Inhibitor (1 U/μl) and 2 μl of PAP enzyme (BioVision M1215-100), in 1× PAP buffer with 2.5 mM MnCl_2_, for 1 hour at 37°C. RNA was purified using the Monarch RNA Cleanup Kit. “Click” reaction with 2 mM DBCO-PEG4-Biotin (Jena Bioscience CLK-A105P4-10) or 2 mM sulfo-Cyanine5 DBCO (Lumiprobe 233F0) and RNA with an incorporated 2′-azido-2′-dATP analog was performed in 10% dimethyl sulfoxide for 1 hour at room temperature. Labeled RNA was purified using RNAClean XP magnetic beads (Beckman Coulter, A63987). Labeling efficiency was verified either in agarose gel run in 1× tris-borate EDTA (TBE) buffer and stained with ethidium bromide (EtBr) or by reversed-phase HPLC. Reversed-phase HPLC RNA samples were analyzed on an Agilent 1260 Infinity HPLC system using an XBridge Oligonucleotide BEH C18 Column, 130 Å, 2.5 μm, 4.6 mm by 50 mm (Waters, 186003953) at 55°C and a linear gradient of buffer B [0.1 M triethylammonium acetate (pH 7.0) and 50% acetonitrile] from 21.9 to 35% in buffer A [0.1 M triethylammonium acetate (pH 7.0)] over 20 min and at a flow rate of 0.9 ml/min.

### dsRNA preparation

Annealing of equimolar amounts of sense and antisense RNA strands was performed as follows: 360 ng/μl of each RNA strands was incubated in RNA annealing buffer [10 mM tris-HCl (pH 7.0), 150 mM NaCl, and 1 mM EDTA] for 3 min in 90°C and then slowly cooled down to room temperature. The efficiency of duplex formation was analyzed in agarose gel run in 1× TBE buffer and stained with EtBr. In addition, formation of dsRNA was verified by RNase I assay as follows: 250 ng of ssRNA or dsRNA was incubated in 10 μl in RNase I buffer [100 mM tris-HCl (pH 7.5), 10 mM NaCl, and 0.1 mM EDTA] with 0.1 U of RNase I (Thermo Fisher Scientific, EN0601) for 5 min at 37°C. Reaction products were analyzed in agarose gel run in 1× TBE buffer and stained with EtBr.

### RNA-seq

A549 cells were seeded in 12-well plate, cultured to 80% confluency, and transfected with dsRNA (350 ng/ml) using 2 μl of mRNA Boost Reagent and 2 μl of TransIT-mRNA Reagent [components of the TransIT-mRNA Transfection Kit (Mirus MIR 2250)] in 100 μl of Opti-MEM (Gibco, 51985026) per 1 ml of cell culture. For mock transfection, cells were transfected with no dsRNA. Four independent replicates were performed. Total RNA was isolated 5 hours posttransfection using TRI Reagent Solution (Invitrogen, AM9738) and RNeasy Mini Kit (Qiagen, 74104). The residual DNA was removed by on-column treatment with DNase I. RNA libraries were prepared by the Genomics Core Facility (RRID:SCR_022718) at the University of Warsaw. Libraries were sequenced on Illumina NovaSeq 6000 with 2 × 150–bp paired-end reads. The raw sequence files were preprocessed using Trimmomatic version 0.39 to trim Illumina adaptor sequences (*82*) and low-quality read fragments. Trimmed sequences were mapped to the human reference genome provided by ENSEMBL v. grch38_snp_tran using Hisat2 (*83*). Optical duplicates were removed using MarkDuplicates tool from GATK package version 4.1.2.0. (*84*). Mapped reads were associated with transcripts from Ensembl GRCh38 database version 100 using HTSeq-count version 0.9.1. (*85*). Differentially expressed genes were selected using DESeq2 package version 1.16.1. (*86*). Fold change was corrected using a normal method. Overrepresentation of GO (*87*) terms among the top 5% of genes (according to *P* value) was assessed with the clusterprofiler package (*88*). RNA-seq data have been deposited at the GEO repository under accession number GSE247857 (password: sfepiaiczxmlzuv).

### dsRNA interactome capture

A549 cells were cultured to 90 to 95% confluency in 100-mm culture dishes and transfected with dsRNA (350 ng/ml) as described above. Three independent replicates were performed. After 5 hours, cells were washed one time in ice-cold phosphate-buffered saline (PBS), and a 100-mm plate with cells covered with 2.5 ml of ice-cold PBS was deposited on a metal plate precooled on ice and irradiated at 150 mJ/cm^2^ with 254-nm UV light in a UVP Crosslinker CL-1000. After cross-linking, cells were detached with the use of a scraper in 600 μl of lysis buffer [50 mM tris-HCl (pH 7.4), 100 mM NaCl, 1% Igepal CA-630, 0.1% sodium dodecyl sulfate, 0.5% sodium deoxycholate supplemented with cOmplete Protease Inhibitor Cocktail EDTA-free (Roche, 4693132001)] per dish. Cells and buffer from each dish were collected and transferred to one Eppendorf-type tube. The mixture was aspirated into a syringe and passed seven times through a 26G needle. Next, lysates were clarified by centrifugation at 10,000*g* at 4°C for 10 min. Ten microliters of each lysate was saved for RT-qPCR analysis. Equilibrated in lysis buffer (buffer was exchanged twice), Dynabeads Protein G beads (Invitrogen, 10004D) were coupled with mouse anti-dsRNA (J2) antibody (1 μg per 20 μl of beads) (Scicons, 10010500) for 1 hour at room temperature with rotation. Then, beads were washed three times with lysis buffer. Lysates were incubated with 30 μl of antibody-coupled beads for 2 hours at 4°C with rotation. Next, beads were washed twice with high salt wash buffer [50 mM tris-HCl (pH 7.4), 1 M NaCl, 1 mM EDTA, 1% Igepal CA-630, 0.1% sodium dodecyl sulfate, and 0.5% sodium deoxycholate] and twice with wash buffer [20 mM tris-HCl (pH 7.4), 10 mM MgCl_2_, and 0.2% Tween 20]. After last wash, the buffer was discarded, and beads were frozen for MS/MS analysis.

Samples for MS/MS were prepared by adding 20 μl of 100 mM ammonium bicarbonate and 2.5 μl of 200 mM tris(2-carboxyethyl)phosphine, vortexing, and then shaking at 10,000 rpm at room temperature for 30 min. This was followed by adding 2 μl of methyl methanethiosulfonate and shaking for an additional 20 min at room temperature. A Trypsin/LysC Mix prepared in 8 M urea and 100 mM ammonium bicarbonate at a concentration of 0.02 μg/μl was added (50 μl per sample). Next, samples were incubated at 37°C with shaking for 4 hours, then supplemented with 300 μl of ammonium bicarbonate, and digested overnight. Acidification was done using 10 μl of 5% trifluoroacetic acid. Peptides were purified using Oasis HLB 96-well plates, vacuum dried, resuspended in 60 μl of 2% acetonitrile and 0.1% trifluoroacetic acid, and then analyzed using an EvosepOne LC-MS setup coupled to an Orbitrap Exploris 480 mass spectrometer. For peptide loading, Evotips C18 trap columns were prepared according to the manufacturer’s protocol, including activation with 0.1% formic acid (FA) in acetonitrile, incubation in 1-propanol, and equilibration with 0.1% FA in water. Samples were loaded with 30 μl of 0.1% FA, and EvoTips were centrifuged at 600*g* for 1 min. Chromatographic separation was performed at 500 nl/min using a 44-min performance gradient on an EV1106 column. Data acquisition was in positive mode, with MS1 at 60,000 resolution and MS2 at 15,000 resolution, targeting the top 40 precursors with a dynamic exclusion of 20 s. Precursors were fragmented in higher-energy collisional dissociation mode with a collision energy of 30%, and the system operated with a spray voltage of 2.1 kV and a capillary temperature of 275°C.

Raw data were analyzed with PEAKS Studio 10.6 64bit Bioinfor (*89*) and searched against UniProt human (78,120 entries) reference proteomes. Fixed modifications: methylthio (methyl methanethiosulfonate) at cysteines; variable: oxidation methionine, acetyl n-term. MS error, 0.1 Da; MS/MS level, 0.2 Da; false discovery rate, 1%; digestion: trypsin semi-specific; max variable PTM per peptide: 3. Protein level analysis was performed using the “Label Free” PEAKS module.

Each group consisted of three biological replicates; the average signal intensity was calculated for every condition. Data were analyzed in such a way as to indicate which proteins coprecipitate with the immobilized decoy in a repeatable and specific manner compared with the given control group.

The ratio of any protein identified and quantified was calculated in relation to the average level in the mock-treated samples normalized to “1.0.” Heatmaps were done in GraphPad Prism. GO analysis was performed using https://geneontology.org (*90*, *91*). Proteomic data have been deposited at PRIDE repository under accession number PXD043620.

### RT-qPCR

A549 cells were seeded in a 12-well plate, cultured to 80% confluency, and transfected with dsRNA as described above. Three independent replicates were performed. After 5 hours posttransfection, total RNA was isolated using TRI Reagent Solution and RNeasy Mini Kit. The residual DNA was removed by on-column treatment with DNase I. cDNA was synthesized using 1 μg of isolated RNA, M-MLV Reverse Transcriptase (Invitrogen, 28025013), and oligo-dT_20_ primer. Ten microliters of cDNA was added to the qPCR mixture containing SsoAdvanced Universal SYBR Green Supermix (Bio-Rad, 1725271) and gene-specific primers, listed in table S1. Reactions were run in triplicate on LightCycler 480 Instrument II (Roche).

Cell lysates (10 μl) from dsRNA interactome capture experiment were diluted with PBS, and total RNA was isolated from them with NucleoSpin RNA Clean-up (MACHEREY-NAGEL, 740948.250). Total RNA was subjected to DNase I treatment in dedicated buffer for 30 min in 37°C and purified again with NucleoSpin RNA Clean-up. cDNA synthesis (400 ng of RNA was used for reverse transcription) and qPCR analysis were performed as above.

### Immunoblotting

Cells were seeded in 24-well plates, cultured to 70% confluency, and transfected as above. After 7 hours (only to study the phosphorylation status of proteins) or 24 hours, cells were lysed with radioimmunoprecipitation assay (RIPA) buffer [50 mM tris-HCl (pH 8.0), 5 mM EDTA, 1% Igepal CA-630, 0.5% sodium deoxycholate, 0.1% sodium dodecyl sulfate, and 150 mM NaCl] or with Luciferase Cell Culture Lysis Reagent (Promega E1531); the latter approach was used to study the phosphorylation status of PKR and eIF2α. Lysates were mixed with Laemmli sample buffer and heated for 5 min at 95°C. Samples were separated by 10% SDS–polyacrylamide gel electrophoresis. Then, proteins were transferred onto a nitrocellulose membrane using the Mini Trans-Blot Cell and Criterion Blotter (Bio-Rad). Proteins on the membrane were stained with Ponceau S buffer, and 1-hour blocking in 5% skim milk in PBS with Tween 20 (PBST) or in 5% bovine serum albumin in tris-buffered saline with Tween 20 (TBST) was performed. Membranes were incubated with the appropriate primary antibody in PBST or TBST overnight at 4°C. After washing with PBST or TBST, membranes were incubated with secondary antibody in PBST or TBST for 1 hour at room temperature. Detection was performed with the use of Pierce Fast Western Blot Kit (Thermo Fisher Scientific, 35065) in an Amersham Imager 600 (GE Healthcare Life Sciences).

Puromycin incorporation assay was performed as described by Schmidt *et al.* (*92*). Cells were seeded in 24-well plates, cultured to 70% confluency, and transfected as above. After 24 hours, puromycin (4 μg/ml) was added to cells 30 min before harvesting in RIPA buffer. Lysates were mixed with Laemmli sample buffer and proceeded as described above. After visualization, proteins on membranes were stained with naphthol blue black solution (0.01%, w/v).

### Pull-down experiment

A549 cells were cultured to 90% confluency and incubated with IFN-α (0 or 200 U/μl; Pestka Biomedical Laboratories, 11200) for 5 hours. For each condition (−/+IFN-α), lysates from two 100-mm culture dishes were prepared. PBS-washed cells were detached with the use of a scraper in 800 μl of lysis buffer [20 mM tris-HCl (pH 7.4), 150 mM NaCl, 2 mM MgCl_2_, 2 mM DTT, and 0.2% Igepal CA-630 supplemented with cOmplete Protease Inhibitor Cocktail EDTA-free] per dish. Cells and buffer were collected and transferred to one Eppendorf-type tube, and this mixture was aspirated into a syringe and passed seven times through a 26G needle. Next, lysates were centrifuged at 10,000*g* at 4°C for 10 min. Lysates from both plates were pooled and then aliquoted. One hundred microliters of each lysate was saved as “input” for further analysis. Each of six aliquot was mixed with variously modified biotinylated dsRNA (no dsRNA added to the control sample) and incubated at 4°C for 1 hour with rotation. Then, prewashed streptavidin magnetic beads (PerkinElmer, CMG-227) were added (10 μl of 50% slurry per sample) and incubated at 4°C for 30 min with rotation. Prewashing of beads comprised three washes with lysis buffer (without protease inhibitors) followed by two washes with wash buffer [20 mM tris-HCl (pH 7.4), 150 mM NaCl, 2 mM MgCl_2_, 2 mM DTT, and 0.2% Tween 20]. After incubation with lysates, beads were washed twice with lysis buffer and twice with wash buffer. Beads and “input” samples were mixed with Laemmli sample buffer and heated for 5 min at 95°C. Samples were separated in 10% SDS-polyacrylamide gel electrophoresis. Then, proteins were transferred onto a nitrocellulose membrane using the Mini Trans-Blot Cell (Bio-Rad). Proteins on the membranes were stained with Ponceau S buffer, and 1-hour blocking in 5% skim milk in PBST was performed. Membranes were incubated with the appropriate primary antibody in PBST overnight at 4°C. After washing with PBST, membranes were incubated with secondary antibody in PBST for 1 hour at room temperature. Detection was performed as above.

### RNase L activity analysis

Cells were seeded in 12-well plates, cultured to 70% confluency, and transfected as above. Alternatively, 5 × 10^5^ cells were electroporated with 5 μg of dsRNA in 100 μl of Ingenio Electroporation Solution (Mirus MIR 50117) in a 0.2-cm cuvette at room temperature using the Ingenio EZporator Electroporation System (Mirus) set to 150 V. Total RNA was isolated 24 hours posttransfection or 2 hours postelectroporation using TRI Reagent Solution and Monarch RNA Cleanup Kit. Total RNA was analyzed in denaturing agarose gel (NBC buffer/formaldehyde) and stained with EtBr.

### UV-VIS measurements

The measurements were performed as described in references (*93*, *94*) with some changes. The UV-VIS measurements was done in cuvettes (1 by 1 cm) containing a 0.5 μM dsRNA in 1 ml of phosphate buffer (10 mM Na_2_HPO_4_, 10 mM NaH_2_PO_4_, and 20 mM NaCl, pH 9.5). Melting temperature determination was performed in a Thermo Fisher Scientific Evolution 300 UV-VIS spectrometer, equipped with a Peltier Thermostatted System and Temperature Probe. The temperature ranged from 16° to 93°C with a ramp rate of 1.0°C/min. RNA duplex unwinding was monitored at 260 nm. All experiments were performed in triplicate. The obtained data were fitted using GraphPad Prism 10.4.1 with the built-in dose response-stimulation (four parameters) function, applying the following constrains: 0 was defined as the smallest mean and 1 as the largest mean value in each dataset. Only absorbance values recorded between 70° and 93°C were used for analysis. The median effective concentration (EC_50_) parameter determined from the fit was interpreted as the duplex melting temperature (*T*_m_).

### Microscopy and image analysis

Cells were seeded on coverslips to reach 60% confluency the next day. Cells were transfected with Cy5-labeled dsRNA (350 ng per 1 ml of culture media) using 2 μl of mRNA Boost Reagent and 2 μl of TransIT-mRNA Reagent (components of the TransIT-mRNA Transfection Kit) in 100 μl of Opti-MEM per 1 ml of cell culture. After 5 hours, cells were fixed with 4% paraformaldehyde at room temperature for 10 min and permeabilized with 0.1% Triton X-100 in PBS at room temperature for 10 min. Next steps were as follows: blocking for 1 hour at room temperature with 5% donkey serum (Sigma-Aldrich, S30) and 0.3 M glycine in PBS; staining overnight using primary antibody at 4°C followed by three washes with PBS; and last, incubation with secondary antibody and 5 U of Alexa Fluor 555 Phalloidin conjugate (Invitrogen A34055) for 1 hour at room temperature. Hoechst 33342 (1 μg/ml; Invitrogen, H3570) in PBS was used to stain the nuclei for 5 min at room temperature, and then cells were washed two times with PBS. Coverslips were mounted using ProLong Diamond Antifade Mountant (Invitrogen, P36965) and imaged using a Carl Zeiss LSM 700 with an Axio Imager Z2 confocal laser scanning microscope, with a 40×/1.3 oil objective. The Hoechst emission, Alexa 488 emission, Alexa 555 emission, and Cy5 emission were detected at emission spectra of 300 to 483, 493 to 550, 560 to 800, and 644 to 800 nm, respectively, after excitation at 405 nm for Hoechst, 488 nm for Alexa 488, 555 nm for Alexa 555, and 639 nm for Cy5. Quantification of foci containing dsRNA or PKR was performed using Arivis Vision4D version 4.1.2. The analysis pipeline included the following operations: denoising noise reduction by Gaussian filtering, intensity threshold, segmentation, size-based filtering, and spatial colocalization of segmented objects.

For the puromycin incorporation assay, cells were transfected as described above. Twenty-four hours post-transfection, puromycin (4 μg/ml) was added for 30 minutes before fixation. The cells were then processed as described above.

### Luciferase assay

A549 cells were cultured to 80% confluency in a six-well plate and transfected with 1.5 μg of IFN-Beta_pGL3 plasmid [gift from N. Manel, Addgene plasmid no. 102597 (*95*)] using Lipofectamine 3000. The following day, transfected cells were seeded on a 96-well plate. After 24 hours, cells were transfected with dsRNA (35 ng per 100 μl of culture media) using the TransIT-mRNA Transfection Kit. Twenty-four hours post-dsRNA transfection, cells were treated with Pierce Firefly Luc One-Step Glow Assay Kit (Thermo Fisher Scientific, 16196) and the luminescence of firefly luciferase was measured in a Synergy H1 (BioTek) microplate reader.
