# Supplementary material for: Effective recognition of double-stranded RNA does not require activation of cellular inflammation
Source: Sci Adv. 2025 Apr 9;11(15):eads6498. doi: 10.1126/sciadv.ads6498 (PMC11980852; doi:10.1126/sciadv.ads6498)
Supplement: Supplementary file 1 — Figs. S1 to S15 Tables S1 and S2 Legends for data S1 and S2 [file sciadv.ads6498_sm.pdf]

Science Advances
AAAS

Supplementary Materials for

Effective recognition of double-stranded RNA does not require activation of cellular inflammation

Karolina Drazkowska *et al.*

Corresponding author: Pawel J. Sikorski, pawelsikorski@uw.edu.pl

Sci. Adv. **11**, eads6498 (2025)
DOI: 10.1126/sciadv.ads6498

The PDF file includes:

Figs. S1 to S15
Tables S1 and S2
Legends for data S1 and S2

Other Supplementary Material for this manuscript includes the following:

Data S1 and S2

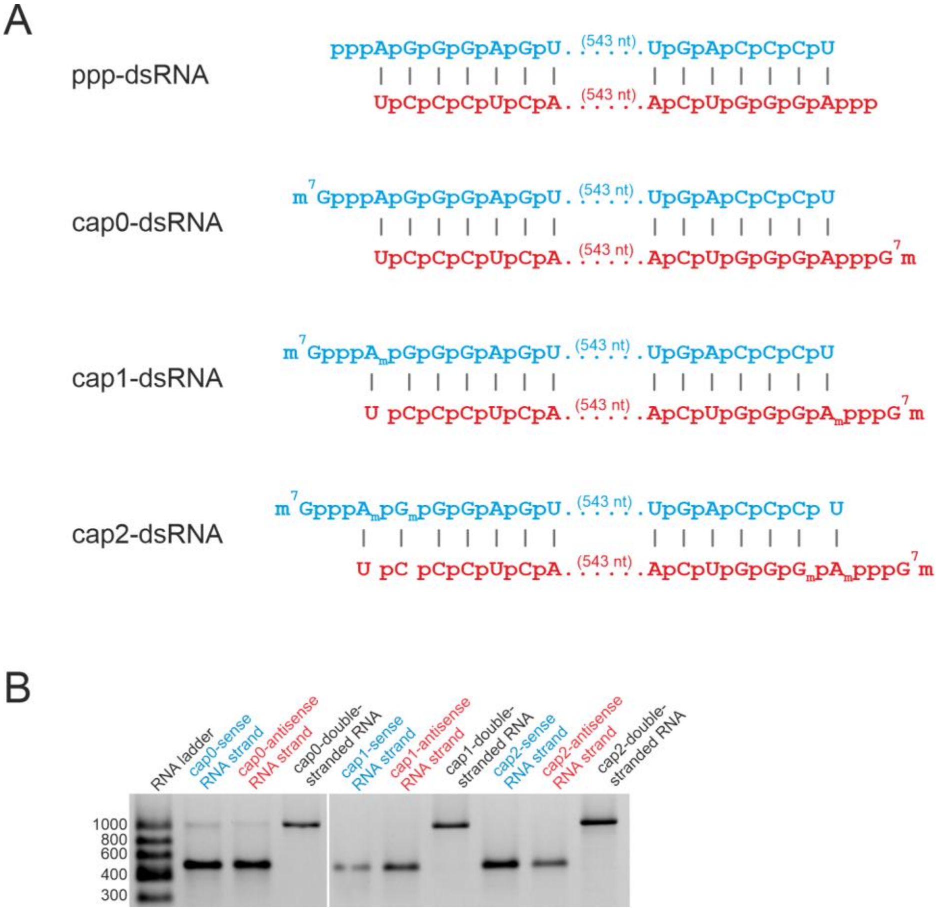

Fig. S1. Preparation of dsRNA. (A) Schematic representation of 5’ end differently modified *in vitro* transcribed dsRNA. (B) Analysis of the sense and antisense dsRNA strands with cap0, cap1, or cap2 at the 5’ end, and duplexes form with these strands on an agarose gel.

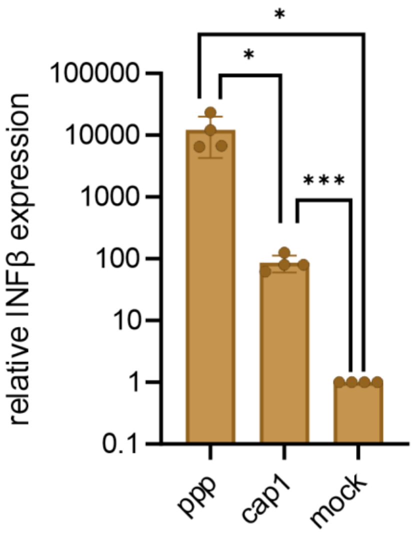

Fig. S2. Activation of IFN pathway upon transfection with dsRNA. Levels of IFNβ in A549 cells after 5 h transfection with ppp- and cap1-dsRNA. Bars represent the mean value of mRNA level change (relative gene expression) ± SD from four independent biological replicates; each independent biological replicate consists of a single transfection reaction. Each point represents data from one independent biological replicate. Data were normalized to mock treated cells. Statistical significance: * $P < 0.05$, *** $P < 0.001$ (one-way ANOVA with Turkey's multiple comparisons test). Only statistically significant differences were marked on the graph. Data were normalized to mock treated cells.

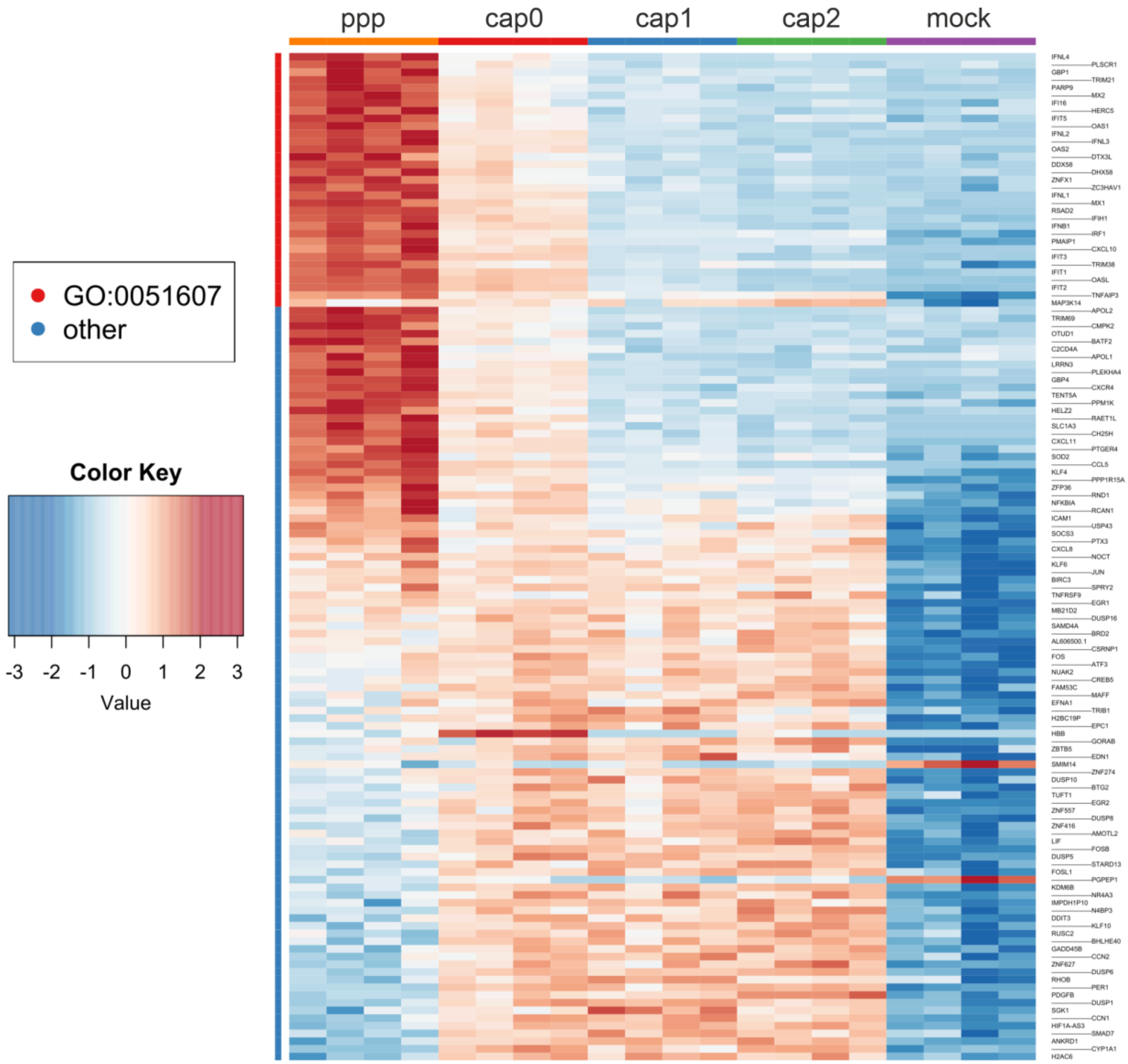

Fig. S3. Comparison of immunogenic potential of dsRNA with differently modified 5' end. Heatmap of top 0.4% upregulated genes for all analyzed conditions (A549 cells were transfected for 5 h with dsRNA carrying different modifications at its 5' ends). Raw RNA-Seq data can be found in the supplemental files.

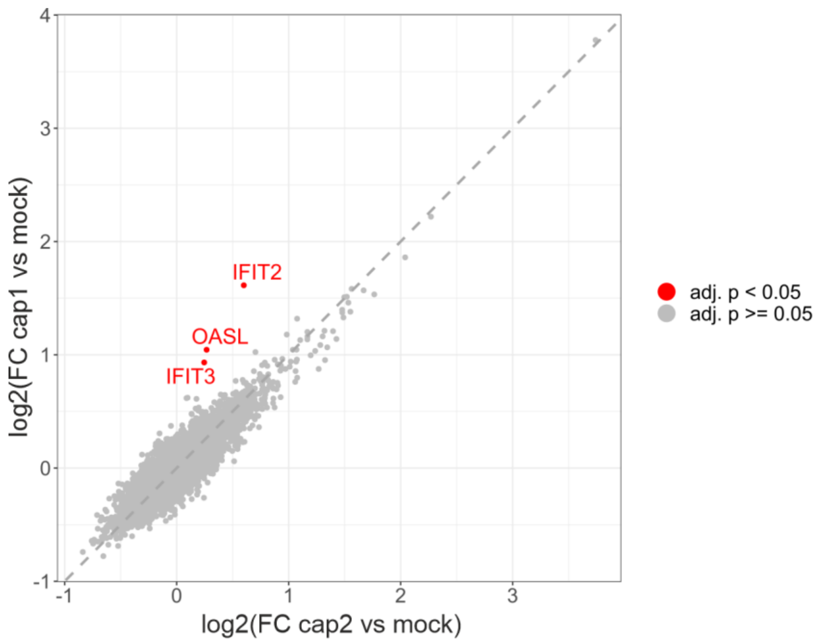

Fig. S4. Comparison of immunogenic potential of cap1- and cap2-dsRNA. Scatter plot of log2-fold change (log2(FC)) upon dsRNA stimulation for 5 h. log2(FC) values in A549 cells transfected with cap2-dsRNA were plotted against those in A549 cells transfected with cap1-dsRNA. Three differentially expressed genes are colored red.

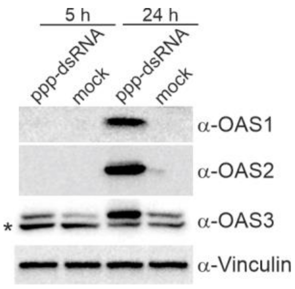

Fig. S5. ISGs expression over time. Comparison of ISG products level (western blotting) in A549 cells after 5 h and 24 h transfection with ppp-dsRNA. * indicates unspecific band.

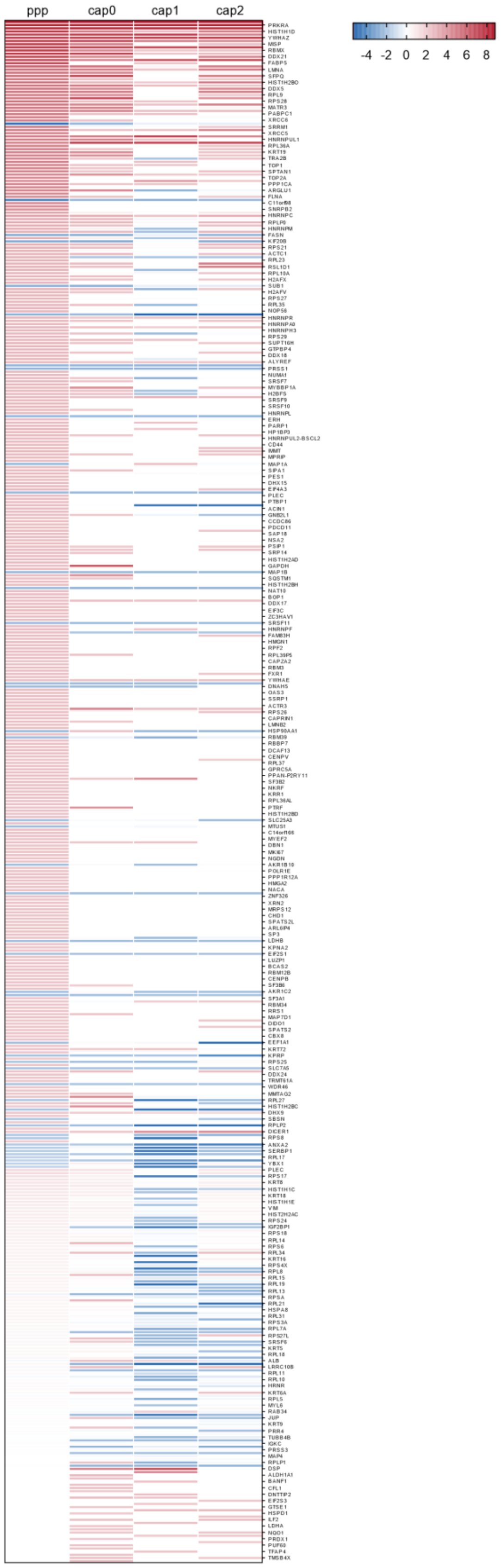

ppp
cap0
cap1
cap2
-4 -2 0 2 4 6 8

Fig. S6. Proteins identified in IPs. Protein specificity heatmap based on the data shown in Fig. S7. This heatmap visualizes the specificity of identified proteins across different experimental conditions. The specificity value was calculated as the log2 intensity ratio compared to the control. All identified proteins with a specificity value greater than 0.0 in at least one of the conditions are shown.

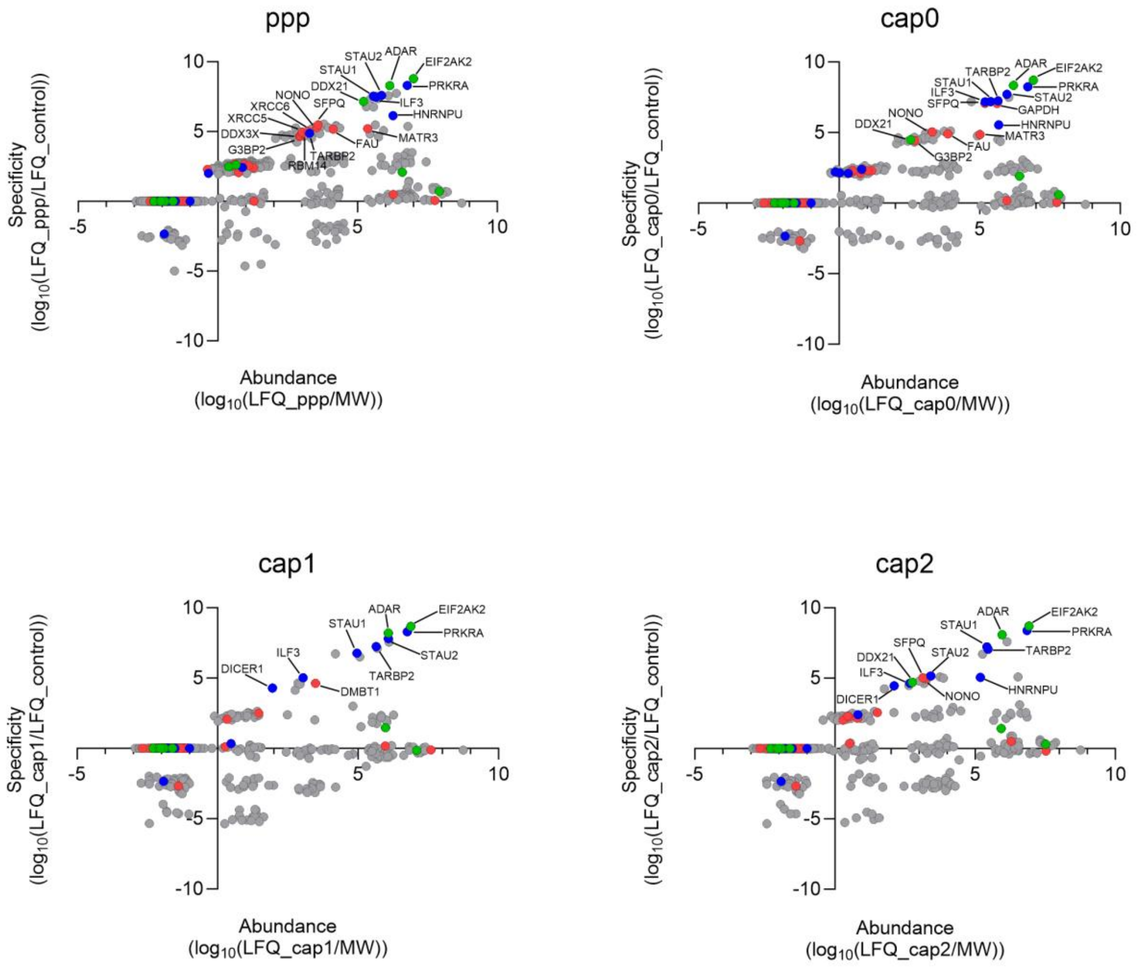

Fig. S7. Interactome of dsRNA with differently modified 5' end. Protein abundance was quantified by normalized signal intensity (LFQ) relative to molecular weight. Specificity (enrichment) was determined by comparing LFQ intensities between dsRNA-transfected A549 cells and mock-treated cells. For proteins not detected in control samples, LFQ was arbitrarily set to 1 for calculations. Proteins annotated as "double-stranded RNA binding" (GO: 0003725) are colored blue, proteins annotated as "innate immunity" (GO:0045087) are colored red, and proteins belonging to both GO terms are colored green.

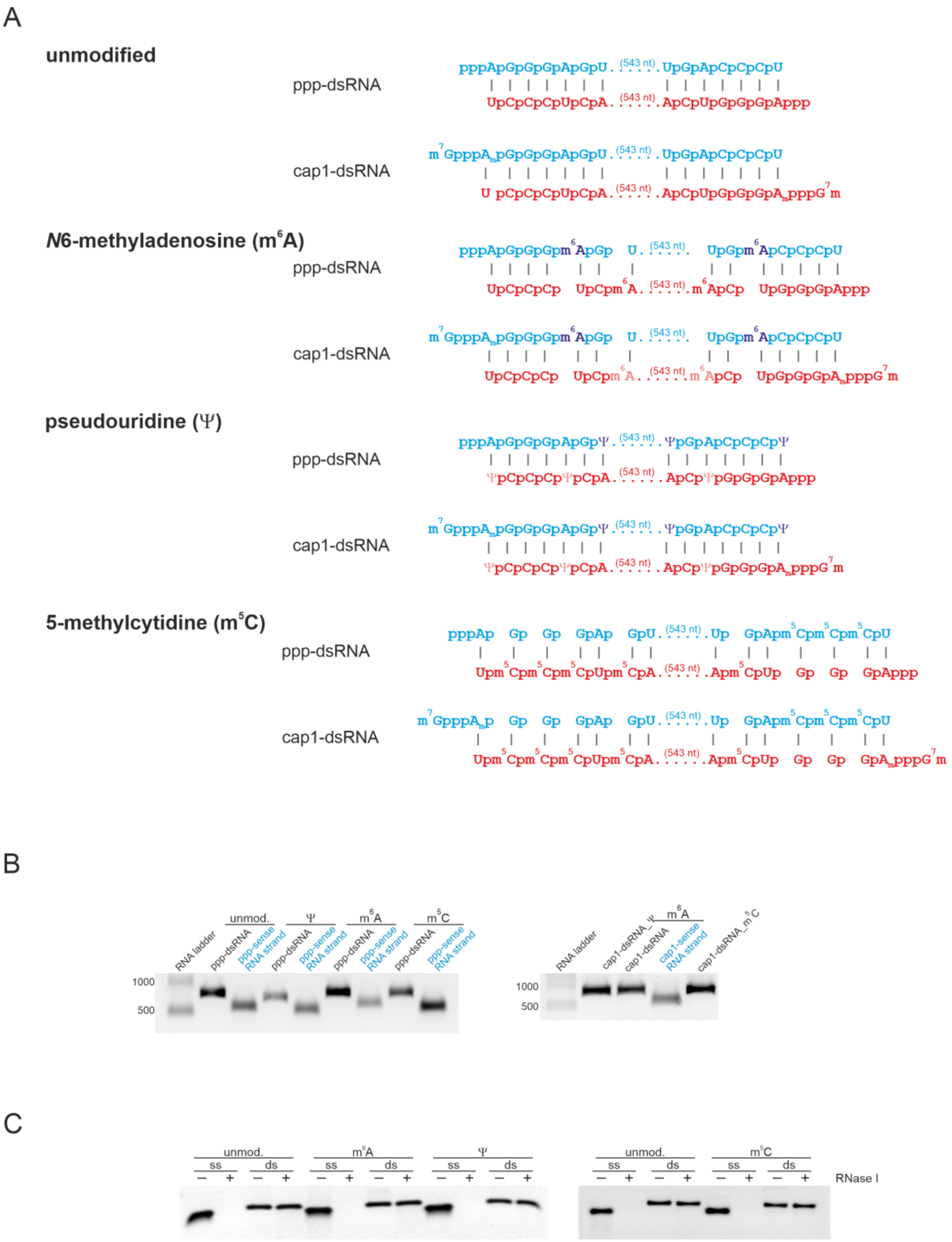

Fig. S8. Preparation of dsRNA bearing epitranscriptomic marks. (A) Schematic representation of 5' end differently modified *in vitro* transcribed dsRNA carrying m^6A, Ψ, or m^5C. (B) Analysis of the sense and antisense dsRNA strands with ppp at the 5' end, and duplexes formed with these strands on an agarose gel. (C) Stability analysis of the generated dsRNA with post-transcriptional modifications using RNase I. Transcripts were incubated with or without RNase I and the resulting products were analyzed on an agarose gel.

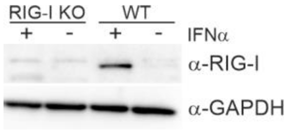

Fig. S9. Verification of RIG-I knockout. Verification of RIG-I knockout in A549 RIG-I KO cells using western blotting. A549(WT) and A549 RIG-I KO cells were incubated with IFNα (200 U/ml) for 24 h and RIG-I expression level was assessed using western blotting.

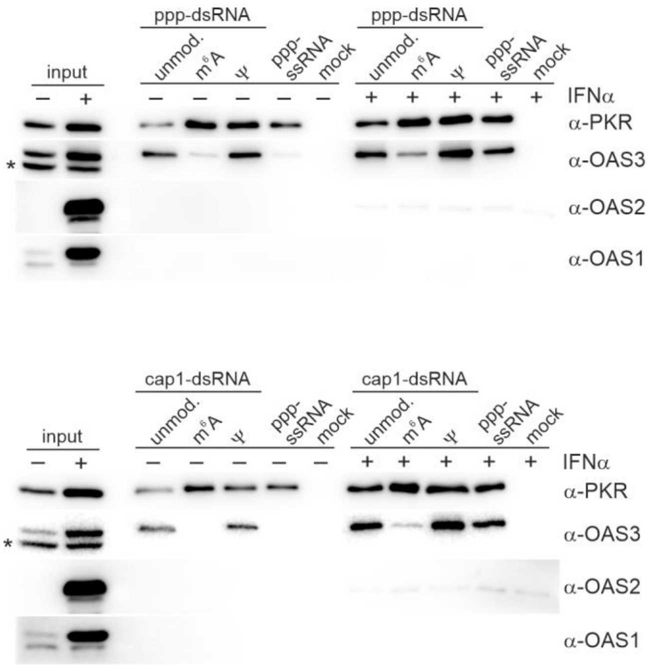

Fig. S10. Proteins identified in pull-downs. Co-purification of endogenous proteins from lysates of IFNα-treated (200 U/ml) and untreated A549 cells with biotinylated ppp- or cap1-dsRNA. PKR, OAS3, OAS2, and OAS1 were detected in precipitates by western blotting. * indicates unspecific band.

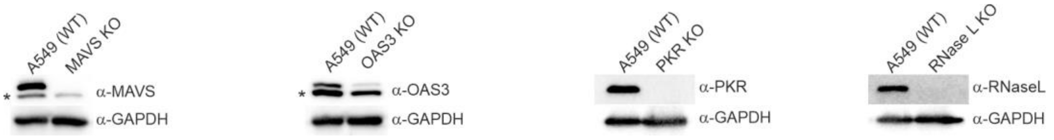

Fig. S11. Verification of MAVS, OAS3, PKR and RNase L knockout. Verification of MAVS, OAS3, PKR and RNase L knockout in MAVS-KO, OAS3-KO, PKR-KO and RNase L-KO cells, respectively, using western blotting. * indicates unspecific band.

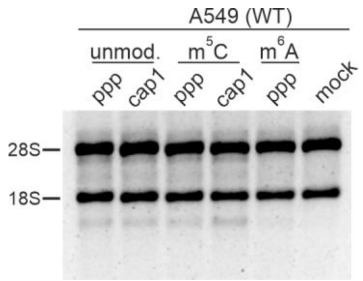

Fig. S12. Presence of m^{5}C does not shield dsRNA from being recognized by OAS/RNase L pathway. The presence of m^{5}C within dsRNA does not affect RNase L activity. RNase L activity in A549 cells was assessed by rRNA integrity. Total RNA was isolated after 24 h transfection with post-transcriptionally modified ppp- or cap1- dsRNA, and analyzed on a 1 x TBE agarose gel.

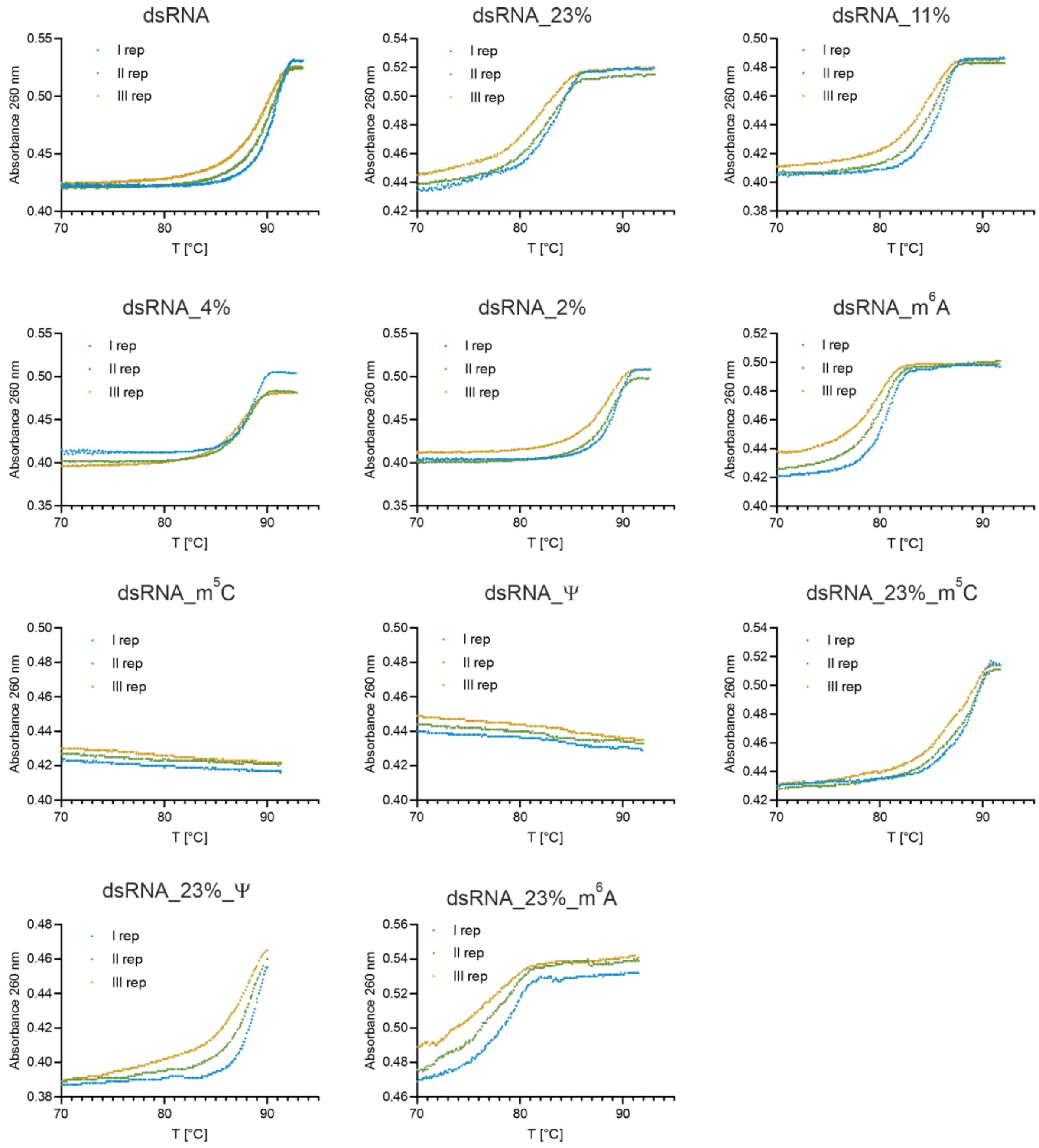

Fig. S13. Thermal stability dsRNA measurements. Raw data from UV-VIS measurements. All measurements were performed in triplicate.

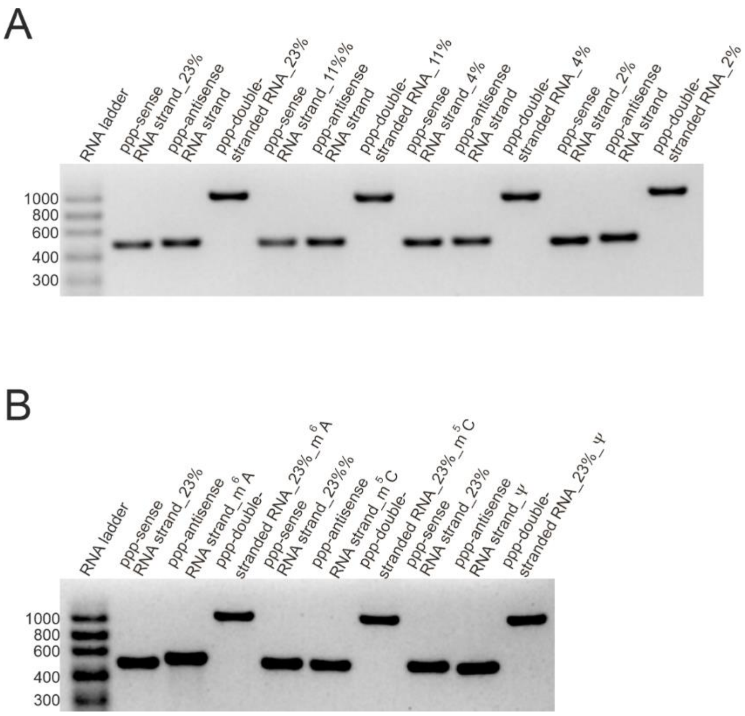

Fig. S14. Preparation of dsRNA for UV-VIS measurements. Analysis of sense strands with varying numbers of adenines replaced by guanines, paired with either (A) unmodified antisense strands or (B) antisense strand bearing epitranscriptomic marks containing a 5’ ppp group, and the duplexes formed with these strands on an agarose gel.

A

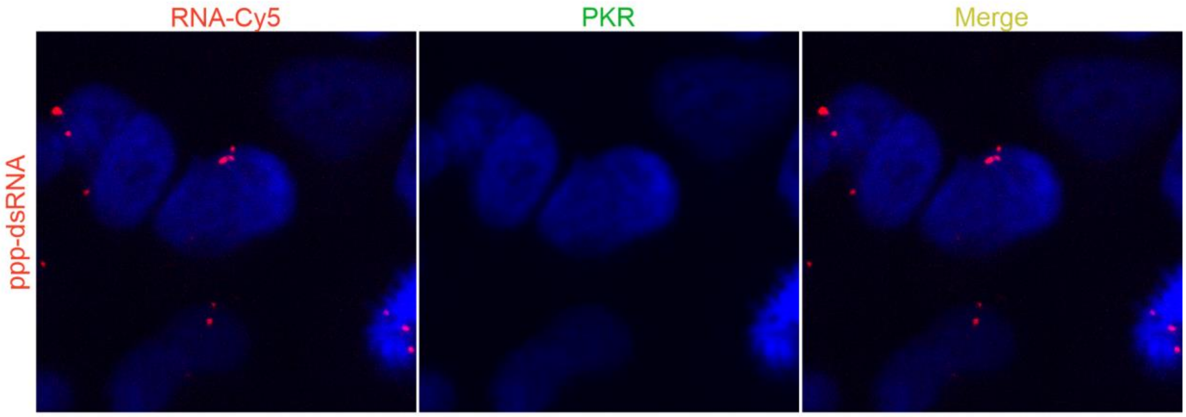

B

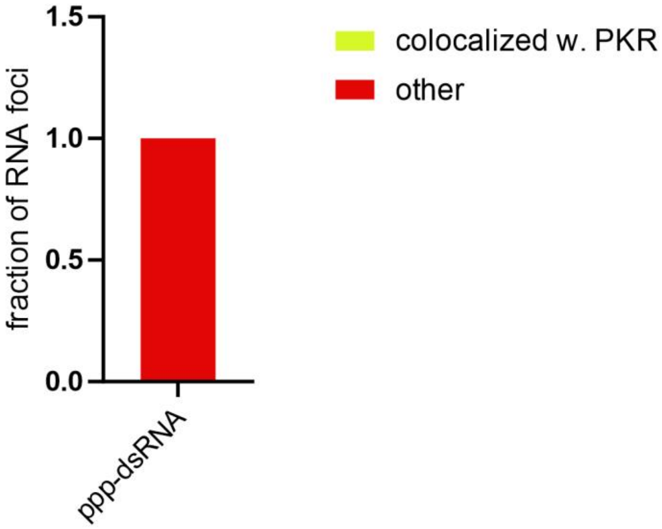

Fig. S15. PKR is dispensable for RNA foci formation. (A) Immunofluorescence analysis of PKR and Cy5-labeled ppp-dsRNA in PKR KO cells. (B) Quantification of Cy5-labeled ppp-dsRNA colocalization with PKR (34 cells were analyzed). Presented bar represents all foci counted, yellow color represents the fraction of foci in which RNA colocalized with PKR, whereas red color represent the fraction of foci in which only signal from PKR was observed.

Table S1.

List of oligonucleotides used in this study.

**Name**	**Sequence**	**Purpose**
GAPDH_for	ACCCACTCCTCCACCTTTGAC	qPCR
GAPDH_rev	TGTTGCTGTAGCCAAATTCGTT	qPCR
IFIT1_for	GATCAGCCATATTTCATTTTGAATC	qPCR
IFIT1_rev	GAAAATTCTCTTCAGCTTTTCTGTG	qPCR
IFIT2_for	AAGAGGAAGATTTCTGAAGAGTGC	qPCR
IFIT2_rev	TCTCCAAGGAATTCTTATTGTTCTC	qPCR
IFIT3_for	GAAGGAACTGGGCCGCCTGCTAAG	qPCR
IFIT3_rev	GCCCTGGCCCATTTCCTCACTACC	qPCR
IFNB1_for	GCCTGGACCATAGTCAGAGTG	qPCR
IFNB1_rev	AGCAATTGTCCAGTCCCAGAG	qPCR
MDA5_for	TTCCGCTATCTCATCTCGTGC	qPCR
MDA5_rev	GGCAGAAAGGTCAGGTAGTCC	qPCR
OASL_for	GTGCCTGAAACAGGACTGTTGC	qPCR
OASL_rev	CCTCTGCTCCACTGTCAAGTGG	qPCR
RIG-I_for	ATGTGCTCCTACAGGTTGTGG	qPCR
RIG-I_rev	ACACTGGGATCTGATTCGCAA	qPCR
Gluc1_for	GCCACGCTGTGTAATACGACTCACTATTAGGG AGTCAAAGTTCTGTTTG	Cloning of sense strand of dsRNA into pJET
Gluc1_rev	CGTCTAGACACCTGCACGTAGGGTCACCACCG GCCCCCTTG	Cloning of sense strand of dsRNA into pJET
Gluc2_for	CGTCTAGACACCTGCACGTAGGGAGTCAAAGT TCTGTTTG	Cloning of antisense strand of dsRNA into pJET
Gluc2_rev	GCCACGCTGTGTAATACGACTCACTATTAGGG TCACCACCGGCCCCCTTG	Cloning of antisense strand of dsRNA into pJET
sgRIG-I_1f	CACCGCGTCATTGTCAGGCACAGAG	CRISPR/Cas9 gene knockout
sgRIG_1r	AAACCTCTGTGCCTGACAATGACGC	CRISPR/Cas9 gene knockout
sgRIG-I_2f	CACCGTGGTTGGACTCGGGAATTCG	CRISPR/Cas9 gene knockout
sgRIG_2r	AAACCGAATTCCCGAGTCCAACCAC	CRISPR/Cas9 gene knockout
sgRIG-I_3f	CACCGGGTGGAGCCTGTGCTGGACT	CRISPR/Cas9 gene knockout
sgRIG_3r	AAACAGTCCAGCACAGGCTCCACCC	CRISPR/Cas9 gene knockout

Table S2.

List of RNAs obtained in *in vitro* transcription reaction.

Name	Sequence
Gluc1	AGGGAGUCAAAGUUCUGUUUGCCCUGAUCUGCAUCGCUGUGGCCGAGGCC<br>AAGCCCACCGAGAACAACGAAGACUUCAACAUCGUGGCCGUGGCCAGCAA<br>CUUCGCGACCACGGAUCUCGAUGCUGACCGCGGGAAGUUGCCCGGCAAGA<br>AGCUGCCGCUGGAGGUGCUCAAAGAGUUGGAAGCCAAUGCCCGGAAAGCU<br>GGCUGCACCAGGGGCUGUCUGAUCUGCCUGUCCCACAUCAAGUGCACGCCC<br>AAGAUGAAGAAGUUCAUCCCAGGACGCUGCCACACCUACGAAGGCGACAA<br>AGAGUCCGCACAGGGCGGCAUAGGCGAGGCGAUCGUCGACAUUCCUGAGA<br>UUCCUGGGUUCAAGGACUUGGAGCCCUUGGAGCAGUUCAUCGCACAGGUC<br>GAUCUGUGUGUGGACUGCACAACUGGCUGCCUCAAAGGGCUUGCCAACGU<br>GCAGUGUUCUGACCUGCUCAAGAAGUGGCUGCCGCAACGCUGUGCGACCU<br>UUGCCAGCAAGAUCCAGGGCCAGGUGGACAAGAUCAAGGGGGCCGGUGGU<br>GACCCU
Gluc2	AGGGUCACCACCGGCCCCCUUGAUCUUGUCCACCUGGCCCUGGAUCUUGCU<br>GGCAAAGGUCGCACAGCGUUGCGGCAGCCACUUCUUGAGCAGGUCAGAAC<br>ACUGCACGUUGGCAAGCCCUUUGAGGCAGCCAGUUGUGCAGUCCACACAC<br>AGAUCGACCUGUGCGAUGAACUGCUCCAAGGGCUCCAAGUCCUUGAACCC<br>AGGAAUCUCAGGAAUGUCGACGAUCGCCUCGCCUAUGCCGCCCUGUGCGG<br>ACUCUUUGUCGCCUUCGUAGGUGUGGCAGCGUCCUGGGAUGAACUUCUUC<br>AUCUUGGGCGUGCACUUGAUGUGGGACAGGCAGAUCAGACAGCCCCUGGU<br>GCAGCCAGCUUUCCGGGCAUUGGCUUCCAACUCUUUGAGCACCUCCAGCGG<br>CAGCUUCUUGCCGGGCAACUUCCCGCGGUCAGCAUCGAGAUCCGUGGUCGC<br>GAAGUUGCUGGCCACGGCCACGAUGUUGAAGUCUUCGUUGUUCUCGGUGG<br>GCUUGGCCUCGGCCACAGCGAUGCAGAUCAGGGCAAACAGAACUUUGACU<br>CCCU
Gluc1_2%	AGGGAGUCAAAGUUCUGUUUGCCCUGAUCUGCAUCGCUGUGGCCGAGGCC<br>AAGCCCGCCGAGAACAACGAAGACUUCAGCAUCGUGGCCGUGGCCAGCAA<br>CUUCGCGACCACGGAUCUCGAUGCUGACCGCGGGGAGUUGCCCGGCAAGA<br>AGCUGCCGCUGGAGGUGCUCAAAGGGUUGGAAGCCAAUGCCCGGAAAGCU<br>GGCUGCACCAGGGGCUGUCUGGUCUGCCUGUCCCACAUCAAGUGCACGCCC<br>AAGAUGAGGAAGUUCAUCCCAGGACGCUGCCACACCUACGAGGGCGACAA<br>AGAGUCCGCACAGGGCGGCAUAGGCGGGGCGAUCGUCGACAUUCCUGAGA<br>UUCCUGGGUUCAAGGACUUGGAGCCCUUGGGGCAGUUCAUCGCACAGGUC<br>GAUCUGUGUGUGGACUGCACAACUGGCUGCCUCGAAGGGCUUGCCAACGU<br>GCAGUGUUCUGACCUGCUCAAGAGGUGGCUGCCGCAACGCUGUGCGACCU<br>UUGCCAGCAAGAUCCAGGGCCAGGUGGGCAAGAUCAAGGGGGCCGGUGGU<br>GACCCU
Gluc1_4%	AGGGAGUCAAGGUUCUGUUUGCCCUGAUCUGCAUCGCUGUGGCCGAGGCC<br>GAGCCCACCGAGGACAACGGAGACUUCAGCAUCGUGGCCGUGGCCAGCAG<br>CUUCGCGACCACGGAUCUCGGUGCUGACCGCGGGAAGUUGCCCGGCGAGA<br>AGCUGCCGCUGGGGGUGCUCAAAGGGUUGGAAGCCAGUGCCCGGAAAGCU<br>GGCUGCGCCAGGGGCUGUCUGAUCUGCCUGUCCCACGUCAAGUGCACGCCC<br>GAGAUGAGGAAGUUCAUCCCGGGACGCUGCCACGCCUACGAAGGCGACGA

	AGAGUCCGCGCAGGGCGGCAUAGGCGGGGCGAUCGUCGACAUUCCUGGGA<br>UUCCUGGGUUCAAGGGCUUGGAGCCCUUGGAGCAGUUCGUCGCACAGGUC<br>GAUCUGUGUGUGGGCUGCACAACUGGCUGCCUCGAAGGGCUUGCCAGCGU<br>GCAGUGUUCUGACCUGCUCAGGAAGUGGCUGCCGCAGCGCUGUGCGACCU<br>UUGCCAGCAGGAUCCAGGGCCAGGUGGGCAAGAUCGAGGGGGCCGGUGGU<br>GACCCU
Gluc1_11%	AGGGAGUCGAGGUUCUGUUUGCCCUGAUCUGCGUCGCUGUGGCCGAGGCC<br>GAGCCCGCCGAGGACGACGGAGGCUUCAGCAUCGUGGCCGUGGCCGGCAG<br>CUUCGCGACCGCGGAUCUCGGUGCUGACCGCGGGGAGUUGCCCGGCGAGG<br>AGCUGCCGCUGGGGGUGCUCAGAGGGUUGGAGGCCAGUGCCCGGAGAGCU<br>GGCUGCGCCAGGGGCUGUCUGGUCUGCCUGUCCCACGUCAGGUGCACGCCC<br>GAGGUGAGGAGGUUCAUCCCGGGACGCUGCCGCGCCUACGAGGGCGACGA<br>GGAGUCCGCGCAGGGCGGCGUAGGCGGGGCGAUCGUCGGCAUUCCUGGGA<br>UUCCUGGGUUCGAGGGCUUGGAGCCCUUGGGGCAGUUCGUCGCACGGGUC<br>GAUCUGUGUGUGGGCUGCACGACUGGCUGCCUCGAGGGGCUUGCCAGCGU<br>GCAGUGUUCUGGCCUGCUCAGGAGGUGGCUGCCGCAGCGCUGUGCGACCU<br>UUGCCGGCAGGAUCCGGGGCCAGGUGGGCAGGAUCGAGGGGGCCGGUGGU<br>GGCCCU
Gluc1_23%	AGGGGGUCGGGGUUCUGUUUGCCCUGGUCUGCGUCGCUGUGGCCGGGGCC<br>GGGCCCGCCGGGGGCGGCGGGGGCUUCGGCGUCGUGGCCGUGGCCGGCGG<br>CUUCGCGGCCGCGGGUCUCGGUGCUGGCCGCGGGGGGUUGCCCGGCGGGG<br>GGCUGCCGCUGGGGGUGCUCGGGGGGUUGGGGGCCGGUGCCCGGGGGGCU<br>GGCUGCGCCGGGGGCUGUCUGGUCUGCCUGUCCCGCGUCGGGUGCGCGCCC<br>GGGGUGGGGGGGUUCGUCCCGGGGCGCUGCCGCGCCUGCGGGGGCGGCGG<br>GGGGUCCGCGCGGGGCGGCGUGGGCGGGGCGGUCGUCGGCGUUCCUGGGG<br>UUCCUGGGUUCGGGGGCUUGGGGCCCUUGGGGCGGUUCGUCGCGCGGGUC<br>GGUCUGUGUGUGGGCUGCGCGGCUGGCUGCCUCGGGGGGCUUGCCGGCGU<br>GCGGUGUUCUGGCCUGCUCGGGGGGUGGCUGCCGCGGCGCUGUGCGGCCU<br>UUGCCGGCGGGGUCCGGGGCCGGGUGGGCGGGGUCGGGGGGGCCGGUGGU<br>GGCCCU

Data S1. (separate file)
MS identification.

Data S2. (separate file)
RNA-Seq data.
